# Host cell transcriptome modification upon exogenous HPV16 L2 protein expression

**DOI:** 10.18632/oncotarget.21817

**Published:** 2017-10-12

**Authors:** Xinwei An, Yuhan Hao, Patricio I. Meneses

**Affiliations:** ^1^ Department of Biological Sciences, Fordham University, Bronx, New York, United States of America; ^2^ Department of Pathology, New York University School of Medicine, New York, New York, United States of America; ^3^ Applied Bioinformatics Laboratories, New York University School of Medicine, New York, New York, United Sates of America

**Keywords:** HPV16 L2, transcriptome modification, RNA sequencing, cell cycle, Rb & Cdc2, Pathology Section

## Abstract

Human papillomavirus type 16 minor capsid protein L2 has been shown to assist in the initial entry and intracellular trafficking events leading to nuclear translocation of the viral genome. During our investigations of L2 function, we observed that expression of L2 in a keratinocyte cell line (HaCaT) resulted in phenotypic changes. In this manuscript, we present data that expression of the L2 protein in this cellular model system HaCaTs resulted in a shift from G0/G1 phase to mitotic S phase, as well as a reduced amount of retinoblastoma protein (Rb) and an increase in Cdc2 phosphorylation. We performed genome-wide host cell mRNA sequencing and identified 2586 differentially expressed genes upon HPV16 L2 expression. Via machine learning and protein network analysis, genes involved in cellular differentiation and proliferation were highlighted as impacted by L2. Our results have implications for the role of L2 at the viral production stages when the virus needs to prevent cellular differentiation while maintaining the cells ability to replicate DNA. Our study suggests a potential novel function of the L2 protein, as a regulator of cellular gene transcription.

## INTRODUCTION

Human papillomaviruses (HPVs) are small, non-enveloped DNA viruses. They infect and replicate in cutaneous and mucosal epithelia [1]. In epithelial tissue, as the basal layer cells migrate to the parabasal layers, cells begin to differentiate and exit the cell cycle. This migration leads to a loss of internal membranes, including the nucleus, and a stoppage of DNA replication [2]. HPVs depend on the cellular replication machinery to achieve their genome amplification, and thus during infection, viral proteins E6 and E7 interact with cellular proteins stimulate and maintain the progression of cell cycle [3]. In these cells the viral genomes are maintained at a low level (50-100 copies per cell) in part to the expression of viral early proteins (E1, E2, in addition to E6 and E7) [4, 5]. There is no viral production in the basal cells, and the ability to replicate DNA in the upper layers of keratinocytes is crucial for amplification of viral DNA and production of viral particles. Prior to or in conjunction with the increase in viral DNA replication in the upper layer, HPV capsid proteins L1 and L2 are expressed. The expression of L1 and L2 are crucial for the completion of viral production as they are necessary to form the capsid, which packages the viral genome.

The minor capsid protein L2 of human papillomavirus type 16 (HPV16) is a critical structural component of the viral particle and is known to be necessary to establish HPV infection [6-8]. After initial binding to receptors, located in extracellular matrix (ECM) or on host cell surface, viral particles go through several conformational changes that expose a buried L2 furin cleavage site [9-14]. Upon furin cleavage, viral particles bind to secondary receptors or a receptor complex that stimulate virion internalization. After entry into host cell is accomplished, a series of vesicle trafficking steps serve as a route for the viral genome to reach the nucleus. Viral genome nuclear import is partly mediated by L2 (and perhaps L1). In addition to L2’s function in viral entry and trafficking, L2 has also been demonstrated to be involved in regulating the immune escape [15].

In an attempt to make a stable L2 cell line for viral production in both 293TT and HaCaT cells, we observed changes in morphology in both cell lines. Thus our attention was diverted towards a potential role of L2 in a cell’s biology. Based on this observation and the knowledge that L2 is expressed at a time when viral life cycle needs to have a dividing cell, we hypothesize that L2 is able to alter cellular transcription to favor viral production. In this manuscript we pursued two lines of research: (1) studies of cell cycle phases distribution and status of key regulators of cell proliferation: cyclin-dependent kinase 1 (Cdk1, also called Cdc2), and tumor suppressor retinoblastoma protein (Rb); and (2) performed a genome-wide host cell transcriptome analysis using mRNA sequencing (mRNA-seq). Our results indicate changes in Cdc2 and Rb expression and phosphorylation, and a significant shift of L2-expressing cells toward mitotic S phase. Gene set enrichment analysis identified significantly modified gene sets that are related to cell proliferation and differentiation. We hypothesize that these changes may have a crucial role in the amplification of viral genome and viral particle production.

## RESULTS

### HPV16 L2 expression drove HaCaTs to S phase and affected Rb and Cdc2 expression and phosphorylation

**Figure 1:**
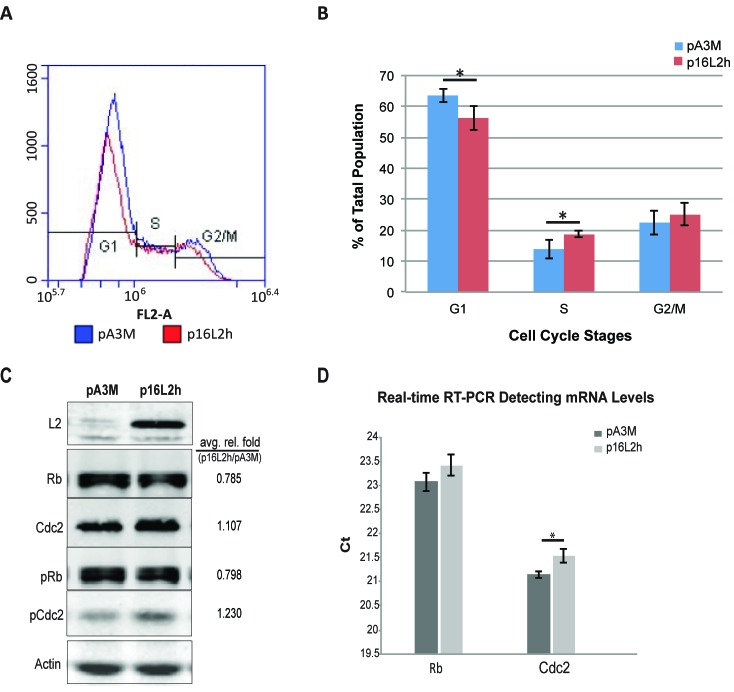
HPV16 L2 expression drives HaCaTs to S phase and affects Rb and Cdc2 expression and phosphorylation Cells were transfected with 500ng p16L2h or empty vector pA3M. 18h after transfection, cells were collected and stained with Propidium Iodide (PI). DNA contents were measured with Accuri^®^ C6 flow cytometer. **A.** Representative results of DNA content detection after empty vector pA3M or p16L2h plasmid transfection. **B.** Average percentage of the cell population in each cell cycle phase. Data are represented as mean±STD from four independent experiments. Significances of difference were analyzed with student’s t-test. *: G1: *p* = 0.0189; S: *p* = 0.0268. **C.** Representative Western Blotting of 500ng plasmid transfection. Average relative fold numbers shown above. Experiments were repeated three times. pRb and pCdc2 indicate phosphorylated Rb and Cdc2, respectively. Protein levels were first normalized to internal control Actin and then calculated as the fold number of pA3M transfected ones. **D.** Two-step real-time RT-PCR of Rb and Cdc2 mRNA levels. Depicted are Ct (Cycle number of threshold) values. Results were compared to the empty vector pA3M transfected group. Data are represented as mean±STD from three independent experiments. Black bars: pA3M transfected cells; gray bars: p16L2h transfected cells. *: *p* = 0.012; student’s *t*-test.

HaCaT cells were transfected with HPV16 L2 (16L2) expressing plasmid p16L2h or empty control vector pA3M and cells were harvested for flow cytometry analysis of cell cycle and Western Blotting analysis of Rb, Cdc2, L2, and β-Αctin levels. Cell population at different phases of cell cycle was determined by staining and measuring DNA content using propidium iodide (PI) flow cytometry. A statistically significant decrease of 10% of cells in G1 phase was observed at 18h after transfection when comparing p16L2h vs. pA3M transfected cells (Figure 1A and 1B). The population of cells in S phase showed a significant increase in the L2 transfected samples.

When comparing cell numbers after transfection, total cell counts dropped after L2 expression (Table 1, total number of cells). The bar graph in Figure 1B shows the average number of population of each phase from three experimental repeats. In summary, these data indicated that 16L2 led to a switch of cell cycle with more cells into S phase.

**Table 1:** Comparison of cell counts among transfection conditions in DNA content detection.

	Total	G1	S	G2/M
pA3M	55920	35425 (63.5%)	7644 (13.95%)	12624 (22.3%)
p16L2h	30048	17024 (56.23%)	5668 (18.75%)	7418 (25.1%)
*p*-value	0.00103	0.000536	0.037238	0.02458

Having observed changes in cell cycle phase distribution, especially the increase of population in S phase, we looked for changes in expression of two cell cycle regulatory genes, Cdc2 and Rb. Cdc2/Cyclin-B complex plays a crucial role in regulating entry into mitosis and is related to cancer development [16]. Inactivation of the complex by either phosphorylation of Cdc2 protein or low level of Cyclin B can lead to cell cycle G2-phase arrest. Rb is a tumor suppressor that plays a negative regulatory role in cell cycle progression and differentiation [17]. It has been proven that Rb is responsible for mitotic cells passing the restriction point and completing the G1/S transition [18].

L2 and A3M transfections were harvested at 18 hours for total protein or RNA. By Western Blotting, we observed a 22% decrease of Rb total protein level after p16L2h transfection (Figure 1C) and a lower amount of Rb mRNAs after 18 hours as measured by real-time RT-PCR (Figure 1D, larger Ct number). In the same experiments, Cdc2 protein level showed an increase (Figure 1C), although a decline in mRNA level (Figure 1D). Because phosphorylation of Rb and Cdc2 are directly related to their functional activity, Western Blotting for phosphorylated Rb and Cdc2 (pRb and pCdc2) was performed. The ratio of phosphorylated to total Rb was not changed, whereas the percentage of phosphorylated Cdc2 increased (pRb/Rb=1.00, pCdc2/Cdc2=1.11; Figure 1C). These results suggest that the increase of total Cdc2 and pCdc2 may contribute to the increase of cells in G2/M phase, and that the decrease of Rb protein abundance might contribute to the increase in S phase population by having more free E2F transcription factor [19].

### Transcriptome analysis revealed 2586 differentially expressed genes upon L2 expression

**Figure 2:**
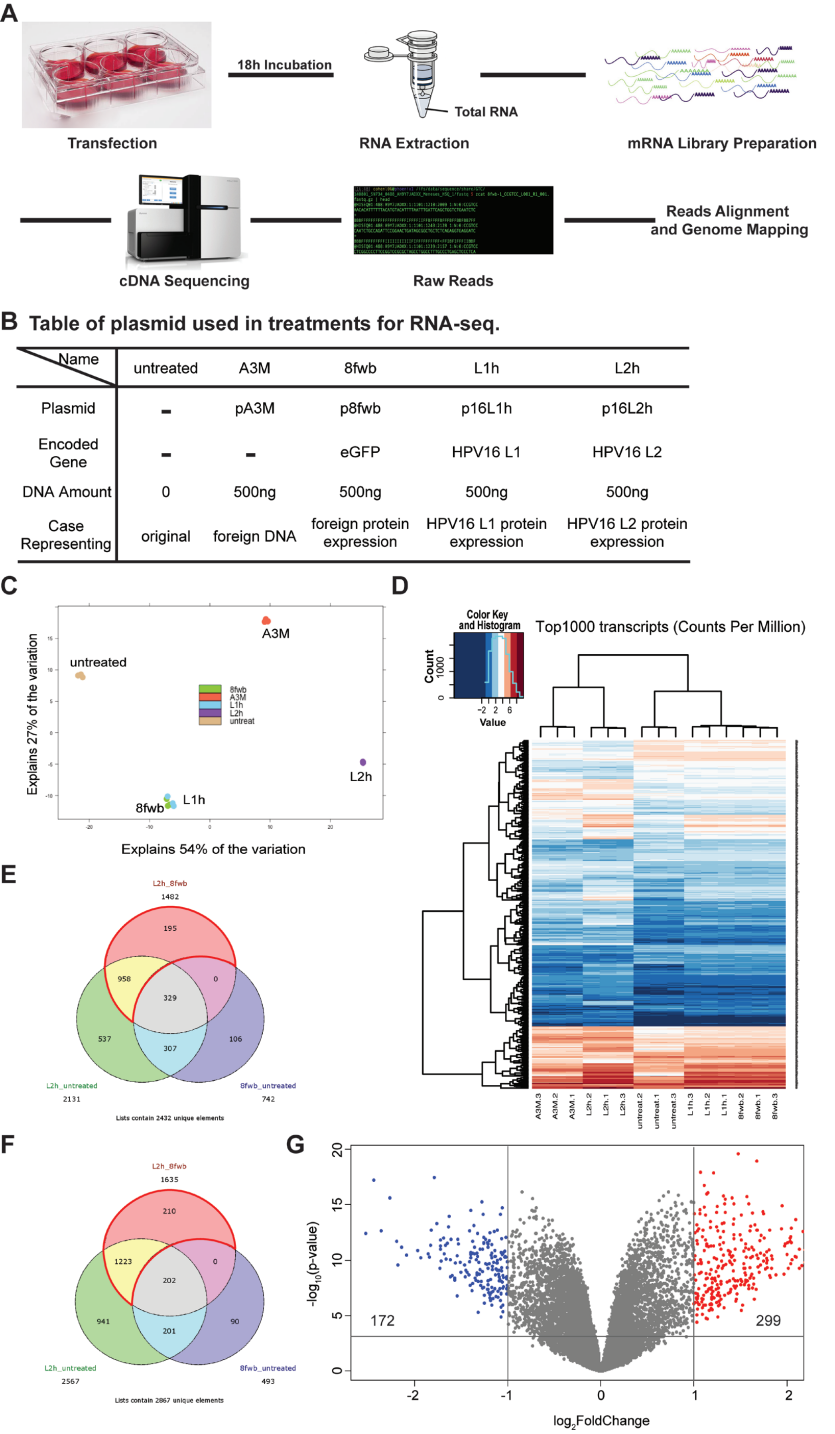
Expression of HPV16 L2 leads to up-regulation of 299 genes and down-regulation of 172 genes HaCaT cells were transfected with 500ng p16L2h plasmid, and total RNAs were collected 18h after transfection. pA3M, p8fwb, p16L1h transfection, as well as untreated HaCaT cell RNAs, were used as the control. **A.** Schematic workflow of RNA-seq and sample preparation. **B.** A table of plasmids used in transfections for RNA-seq sample preparation. **C.** Principle Component Analysis (PCA) result with replicates from each group. **D.** Heatmap of top1000 most abundant variant transcripts among each replicates. **E.** and **F.** Venn diagram showing significantly regulated genes in different comparison conditions. Area enclosed by red lines indicates uniquely affected genes. E: Positively regulated genes in p16L2h transfection; F: Negatively regulated genes in p16L2h transfection; G: Volcano plot of differentially expressed genes (DEGs) in 16L2h:8fwb contrast. Vertical lines denote fold changes greater than ± 2-fold. Horizontal line denotes *p*-value > 0.001. Blue dots indicate genes down-regulated in p16L2h transfected group, and red dots indicate genes up-regulated.

In order to explore if genome-wide transcription changes occur after L2 expression, host cell mRNA sequencing was performed. A schematic workflow of the RNA-seq strategy is shown in Figure 2A (described in Materials and Methods). In our RNA-seq screen we had five experimental conditions on HaCaTs (Figure 2B): untreated cells, or cells transfected with empty vector pA3M, eGFP ORF expressing control plasmid p8fwb, HPV16 L1 ORF expressing plasmid p16L1h, or p16L2h. Cells were transfected in 6-well plates, and total RNAs were collected at 18h post-transfection. RNA sample quality was assessed before preparing mRNA library. Using poly(A) tail as bait, mRNAs were purified and used for next generation sequencing (NGS) library preparation. Two rapid single-read 50 Illumina HiSeq sequencing runs were performed, raw reads from separate lanes of the same sample were merged, aligned, and mapped to human genome. Normalized gene expression counts of each RNA-seq sample and sample alignment statistics can be found in Supplementary Table 1.

We first used Differential Gene Expression (DGE) analysis to reveal genes that were up- or down- regulated in their mRNA levels (Differentially Expressed Genes, or DEGs). An adjusted p-value<0.001 was used as the cut-off for the statistic significance. To visualize RNA-seq variations and transcript expression patterns in different groups, we made use of both heatmap and the principal component analysis (PCA). By default, the two dimensions that explain most variation in the data set were chosen as x-axis and y-axis. For our data set, the first two principal components explained a total of 81% (54%+27%, shown in Figure 2C) of the variation. This is to say that the most differences across groups observed in our dataset can be explained and represented by PCA results. As a standard analysis means to visualize high dimensional data, PCA showed the comparison results among all five groups and exhibited the differences across groups with their loci in the grids. In Figure 2C, each replicate from the various conditions is represented by a colored dot. Three dots in the same color represent replicates of the same group and indicate three individual transfections and sample collections. The triplicates from each group all mapped to the same location in the grids; thus reproducibility of each condition was demonstrated. The process of DNA transfection discriminated between transfected cells and untreated cells. Expression of foreign protein differed groups 8fwb, 16L1h, and 16L2h from group A3M (the DNA control, no-detectable exogenous protein). In total, PCA results visualized the differences among groups by showing their loci in the grids and explained 81% of the variation observed in our dataset.

With the confirmation that different experimental groups would show different loci in the PCA grids, it is interesting to see that the samples expressing eGFP or HPV16 L1 (8fwb and 16L1h group, respectively) clustered together. This is in contrast to the samples that expressed L2 protein. 16L2h samples clustered far separately from other groups. This suggested that the expression of proteins L1 and eGFP had similar effect to each other, and distinct from 16L2h or untreated or A3M samples. To confirm the expression of the experimental ORFs in the samples, PCA result was then assessed by pseudo-genome mapping, which used eGFP, L1h, and L2h gene coding sequences as a reference pseudo-genome for reads alignment (Supplementary Figure 1). As designed, L2 expression was detected only in the group transfected with p16L2h. This analysis supported that in each transfection group, expression of plasmid-based exogenous gene occurred (Supplementary Figure 1, A, B, and C). Having the reliability of our assay proven, the PCA results of the similarity between 8fwb group and 16L1h group strongly suggested that regarding gene transcription profile, 16L1 protein had no different effect as eGFP. This novel finding suggests that L1 has no observable effect on target cell’s transcription profile and thus no effect on cellular protein networks.

A heatmap was used to visualize the top 1000 most abundant transcripts (counts per million) across all samples (Figure 2D). Using read counts rather than DEGs in a heatmap gave us an unbiased perspective by looking at how much transfected cells tend to vary across samples. In Figure 2D, repeats within each group showed similar color pattern across genes, which were clustered on the left side of the heatmap, as well as apparent differences between groups. For instance, if three repeats of p16L2h transfection showed strong similarity for a certain gene, same or similar color would be found for the same gene (same row) in all 16L2h repeats (three lanes), whereas a gene might have different color in the lane of L2h.1 versus the lane of 8fwb.1.

PCA results demonstrated that changes in 8fwb group were similar with 16L1h group, whereas 16L2h group showed large difference from these two groups as well as untransfected cells or control DNA transfection samples (A3M group). The same trend was observed in the heatmap. These two analyses suggested that 16L2 protein played a role that cannot be fulfilled by any foreign protein since eGFP and 16L1 certainly did not shed the same influence as L2 on the cellular transcriptome, nor due solely to the introduction of plasmid DNA.

GFP has been used in this and many experiments as a control protein, i.e., without biological effect. Our data suggest that introduction of eGFP does result in changes of gene expression when compared to vector transfection (A3M) but equal to HPV16 L1 protein that has also not been implicated as having a biological effect on cellular transcription. We thus compared p16L1h and p16L2h transfected cells with p8fwb transfected group (16L1h:8fwb contrast, or 16L2h:8fwb contrast), and the lists of significantly regulated genes and their Differential Gene Expression (DGE) analysis statistics can be found in Supplementary Table 2. We show the varying genes in analyses using Venn Diagrams (Figure 2E and 2F).

In Figure 2E, the pink circular area represents genes that had more transcripts in p16L2h transfected cells as compared to 8fwb transfections. Green area shows up-regulated genes in p16L2h-transfected cells, comparing to untreated HaCaTs. Blue area indicates up-regulated genes in 8fwb plasmid transfected cells, as compared to control HaCaT cells. In Figure 2E, genes that were in the pink circle but not in the blue circle (958+195, in the area enclosed by red arcs) were genes whose up-regulation was caused by p16L2h but not p8fwb, i.e. by 16L2 protein but not eGFP. Genes in 16L2h group that showed variance on their mRNA level when compared to the 8fwb group (16L2h_8fwb comparison) but not any differential expression in the 8fwb_untreated comparison were selected as uniquely regulated genes. Unique genes were listed in Supplementary Table 3. Down-regulated genes were shown in Figure 2F.

Our transcriptome analysis identified 1153 genes positively regulated and 1433 genes negatively regulated at their mRNA levels in L2 expression samples. We also did the same analysis for 16L1h group, and one gene, *rpl12*, was identified as the unique gene. *Rpl12* was negatively regulated in the L1 expression examples, while no positively regulated gene was detected. *Rpl12* gene encodes the ribosomal protein L12, a component of ribosome 60S subunit. We did not observed any morphological changes in our cultures expressing L1 or eGFP. Hence, we theorize they have no or limited effect on cell biology, i.e., irrelevant to cell function.

To probe more deeply to the influences that 16L2 can shed on HaCaTs transcriptome, using more restricted cut-off settings, we narrowed the differentially expressed genes (DEGs) to 471 (p-value < 0.001 and |logFC| > 1). Statistics of all results from the DGE analysis of duplicates of RNA-seq data were used. Particularly, p-value and logFC of each DEG were transformed and then exhibited in the volcano plot (Figure 2G). Blue and red dots indicate DEGs that were ±2-fold differentially expressed between L2 and eGFP expressing HaCaTs (299 up-regulated genes, shown as red dots in Figure 2G; and 172 down-regulated genes, shown as blue dots in Figure 2G). The total number of blue and red dots was 471.

### Confirmation of transcriptome findings

To confirm our findings in RNA-seq, we picked out five genes that play important regulatory roles in cell growth, mitosis, and cell proliferation to conduct both real-time RT-PCR and Western Blotting. HaCaTs were transfected with either p8fwb or p16L2h plasmid DNA, and cells were harvested for RNA or total protein preparation. Then the mRNA level and protein level of Cdk6, TGFβ2, MAPK1, FAK, and Pyk2 were analyzed. Levels of both mRNA and protein of these genes are shown in Supplementary Figure 2. The validation confirmed our RNA-seq results.

### Identification of genes and gene sets modified by L2 expression

DGE analysis revealed 2586 significant genes, and with the more restricted cut-off, we narrowed the dataset down to 471 genes. Using these 471 genes, we performed computational and statistical analysis in two separated tracks:

1) The first track of analysis included GSEA + LEA and IPA. GSEA and LEA together identified individual genes affected by L2 expression and participated in the regulation and control of cell proliferation and apoptosis. IPA from a pathway analysis angle provided evidence of biological processes that participate in the regulation of cell proliferation and apoptosis were altered upon L2 expression.

2) The second track of analysis included Machine Learning and PANTHER analysis. Using Support Vector Machine (SVM) and Random Forest (RF) for the classification between 8fwb and 16L2h, we further selected 50 genes that were most affected by L2 expression and investigated whether they are functionally related using PANTHER. Our results showed strong support to our hypothesis that it is because pathways and biological processes are altered by L2, that the occurrence of shift of cells from G0/G1 phase to S phase, as well as the change of total cell number.

### 1). Gene Set Enrichment Analysis (GSEA) and Leading edge analysis (LEA) identified cell proliferation and apoptosis regulatory gene sets altered by 16L2 expression

To gain a deeper understanding of cellular transcriptome changes upon 16L2 expression, Gene Set Enrichment Analysis (GSEA) was performed. GSEA software [20, 21] and molecular signatures database (MSigDB) were used for this analysis to determine prior-defined sets of genes that showed statistically significant, concordant differences between L2h group and 8fwb group. Our analysis detected 102 positively regulated and 246 negatively regulated gene sets using a 16L2h:8fwb comparison. These gene sets and their GSEA statistics are provided in Supplementary Tables 4 and 5. Normalized p-value and false discovery rate (FDR) q-value were used to determine gene sets’ statistical significance (p<0.05, and q<0.1, respectively). Hyperlinks to MSigDB are also provided in Supplementary Tables 4 and 5 for detailed information of identified gene sets.

**Figure 3:**
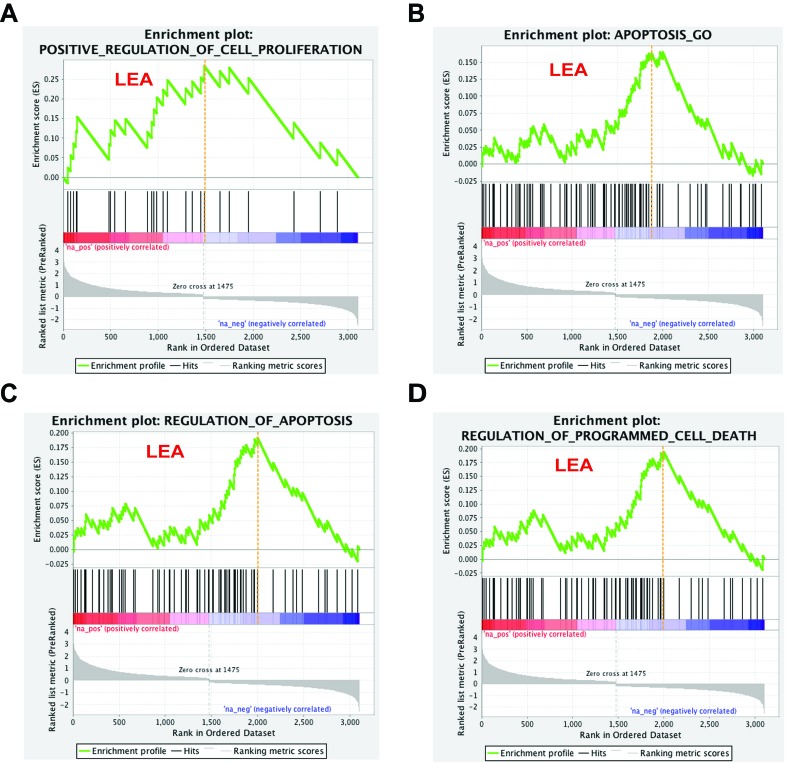
Cell proliferation and apoptosis related gene sets response to 16L2 expression Enrichment plots for four selected gene sets are shown as above. Gene sets are selected based on their biological functions, GSEA *p*-values, and FDR q-values (*p* < 0.05, and FDR<0.1). Green line indicates accumulative enrichment score; black lines show ranking location of genes with gene set; gray color visualizes ranking metric scores; red and blue gradient colors suggest a positive or a negative regulation, respectively; and hits on the left side of orange dash lines are considered within leading edge subset (labeled LEA).

**Figure 4:**
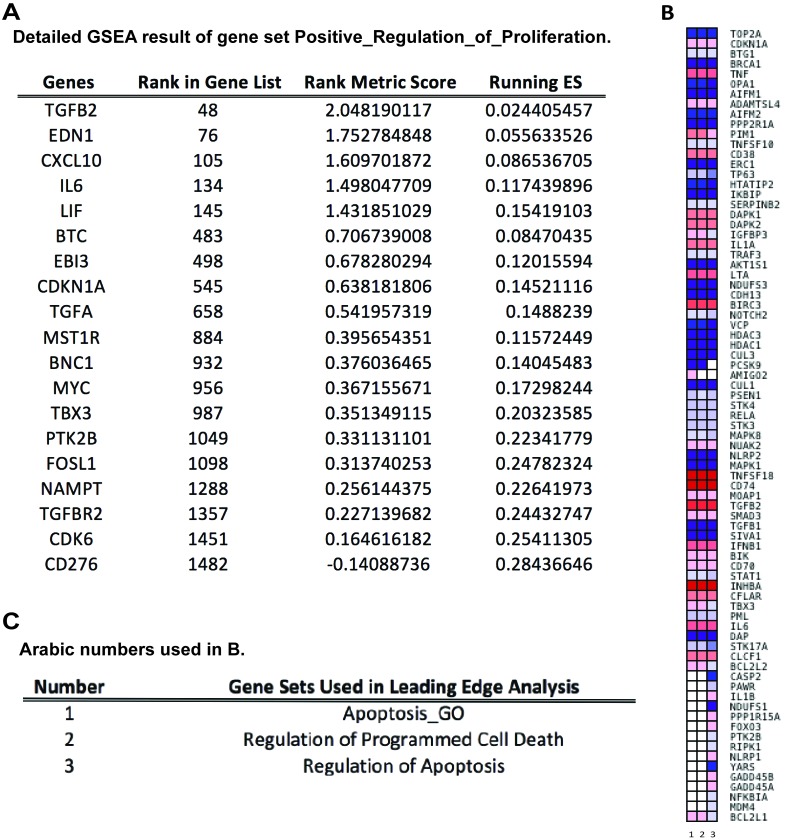
Leading Edge Analysis (LEA) reveals core genes that account for the gene set’s enrichment signal, as well as genes that are overlapped among gene sets **A.** Table of detailed GSEA results of enriched gene set “Positive Regulation of Cell Proliferation”. ES: Enrichment Score. **B.** Heatmap of clustered genes in the leading edge subsets of gene sets related to apoptosis and programmed cell death. Red and blue colors indicate positively and negatively regulated transcripts, respectively, upon HPV16 L2 protein expression. Darker the color, greater the fold difference. Rows are genes in leading edge subsets; columns are gene sets, which are labeled as 1 to 3. **C.** Table of explanations for Arabic numbers used in B.

To pursue the question the effect L2 expression may have on regulation of cell proliferation, and to understand the drop of cell counts in DNA content detection, we looked for enriched gene sets that participate in cell cycle/cell proliferation regulation and programmed cell death. Our analysis demonstrated that the MSigDB prior-defined gene set Positive_Regulation_of_Cell_Proliferation met the strict criteria of both p-value and q-value and was identified as significantly altered by 16L2 expression. Three gene sets, Apoptosis_GO, Regulation_of_Apoptosis, and Regulation_of_Programmed_Cell_Death, with genes participate in apoptosis or programmed cell death satisfied the selection criteria. In the enrichment plots of these four gene sets, red and blue gradient colors were used to represent up- or down-regulated genes, respectively (Figure 3). Accumulative enrichment scores, which reflected the degree to which a gene set was overrepresented at the top or bottom of a ranked list of genes, were indicated by a green line. The score furthest from 0.0 on green line was the enrichment score (ES) for the gene set. Vertical black lines in the middle portion of plots showed where the members of the gene set appear in the ranked list of genes. An orange dash line in each plot was used to locate genes considered within leading edge subset (for gene sets having positive ES, prior to orange dash line), which was a subset of gene in the set that contributed the most to ES of the set. All four gene sets showed positive accumulative ES. Genes identified in the leading edge of these four gene sets were shown in Figure 4A and 4B. These results further highlighted that 16L2 expression up-regulated the positive regulation of cell proliferation. This finding also strongly indicated that L2 expression altered the host cell apoptosis status, and influenced progression into the cell cycle towards mitosis.

To compare the members of the three gene sets that were related to apoptosis regulation, a leading edge analysis (LEA) was done and clustered genes in leading edge subsets are shown in a heatmap (Figure 4B). Colors represented expression values of each gene, while the range of colors showed the range of expression values. The columns in Figure 4B were the selected gene sets. The overlap between the leading edge subsets can be easily recognized as genes across columns colored similarly. As shown in Figure 4B, a majority of these genes had overlapping expression modification and with similar range, indicating that apoptosis and its regulation was a pathway that was strongly affected by HPV16 L2. Genes that overlapped through gene sets and were greatly changed on its regulation may be the most promising candidates for future mechanism studies. Given that only one gene set that was related to cell proliferation regulation was selected by GSEA, LEA was not applied to it. Detailed GSEA statistics of members of this gene set were provided in Figure 4A.

### IPA revealed pathways and up stream regulators that are involved in regulation of cell proliferation affected by L2 expression

To address the question as to what pathways related to cell proliferation were potentially affected by the expression of 16L2, we utilized the core analysis of Ingenuity Pathway Analysis (IPA, Ingenuity Systems, http://www.ingenuity.com/) on our data. The top IPA results based on p-values for the 471 DEGs are summarized in Table 2. All top IPA results, including the top pathways enriched in those DEGs, top activated regulators inferred from those DEGs, top diseases, and top molecular and cellular functions revealed the involvement of those DEGs were related to cellular function in cell growth, proliferation, and cancer biology. HMGB1 (high mobility group box 1) signaling pathway was highlighted by IPA, and 15.3% genes function in this pathway overlapped with our 471 DEGs (20 out of 131, Table 2). This pathway plays important regulatory roles in several cellular processes, including cell differentiation, tumor cell migration [22-24]. Moreover, the upstream regulators predicted by IPA as activated all participated in the regulation of cell proliferation or apoptosis. These include cytokines tumor necrosis factor (TNF) and interleukin 1-alpha (IL1A) which both are involved in the regulation of a wide spectrum of biological processes. Through the interaction with its receptors, TNF regulates cell proliferation, differentiation, apoptosis, etc. Active IL1A can be released by its proteolytic process in response to cell injury, and thus induces apoptosis. Also included, RELA (RELA Proto-Oncogene, NF-κB Subunit. Also known as p65; NFκB3) directly bind with free NFκB upon the degradation of inhibitor of NFκB and forms the active complex for NFκB to fulfill its function as a ubiquitous transcription factor; protein encoded by Jnk gene is a member of MAP kinase family. MAPKs act as an integration point for multiple biochemical signals that are also involved in proliferation, differentiation, and transcription regulation [25].

**Table 2:** Top IPA results for the 471 differential expressed genes (DEGs) identified from the 16L2h:8fwb contrast.

Top Canonical Pathways	*p*-value	Overlap*
Role of Macrophages, Fibroblasts and Endothelial Cells in Rheumatoid Arthritis	3.26E-14	11.3%, 34/300
HMGB1 Signaling	3.15E-11	15.3%, 20/131
Hepatic Cholestasis	8.73E-10	12.7%, 20/157
Role of Osteoblasts, Osteoclasts and chondrocytes in Rheumatoid Arthritis	8.80E-10	10.6%, 24/227
Hepatic Fibrosis / Hepatic Stellate Cell Activation	1.87E-09	11.6%, 21/181
**Top Upstream Regulators**	***p*-value of overlap**	**Predicted Activation**
TNF	4.29E-20	Activated
IL1A	8.23E-14	Activated
RELA	1.73E-12	Activated
Jnk (MAPK8)	1.60E-10	Activated
**Top Diseases and Disorders**	***p*-value range**	**# Molecules**
Cancer	9.76E-04 - 4.36E-13	428
Organismal Injury and Abnormalities	9.78E-04 - 4.36E-13	434
Dermatological Diseases and Conditions	5.80E-04 - 1.95E-12	267
Reproductive System Disease	9.69E-04 - 1.55E-10	262
Inflammatory Disease	9.31E-04 - 1.09E-09	105
**Top Molecular and Cellular Functions**	***p*-value range**	**# Molecules**
Cell-To-Cell Signaling and Interaction	9.76E-04 - 1.83E-12	89
Cellular Development	9.76E-04 - 4.45E-12	134
Cellular Movement	8.70E-04 - 1.26E-11	96
Cellular Growth and Proliferation	9.76E-04 - 3.74E-11	148
Lipid Metabolism	8.38E-04 - 1.53E-08	50

*Overlap: genes shared between 471 DEGs and genes in a canonical pathway

### 2) Selection of genes using Machine Learning and their functional clustering with PANTHER

We have shown that in HaCaTs, 2586 genes expression profile has been significantly affected by the 16L2 expression, and 471 genes among them were selected by computational analysis as more convincing candidates, for a higher level of changes of the mRNA abundance (|logFC|>1). Gene set enrichment analysis, as well as protein functional and clustering analysis, led our attention to those participate in cell cycle progression and regulation. These gene sets and protein clusters revealed pathways and regulators that play important roles in the control of cell grow and dividing were affected by the expression of 16L2. These findings also provide strong supports to our hypothesis that the changes in cell morphology we observed were a direct result of L2 expression.

To validate our findings of genes and pathways that directly participate in or strongly related to cell cycle progression or regulation that were affected by 16L2 on their mRNA expression, we conducted two machine learning feature selection models: Support Vector Machine (SVM) and Random Forest (RF, details in Materials and Methods). The 471 DEGs selected from the 16L2h:8fwb comparison were analyzed. The feature selection model assigns each gene an importance ranking which is calculated based on optimization of classification accuracy. To avoid over-fitting issues and model bias, two different models SVM and RF were applied, and then we combined both importance rankings together to obtain the merged gene ranking list (Supplementary Table 6, top 50 genes with ranking scores). The gene with a higher ranking is deemed to be more important for the classification of L2h and 8fwb. The results indicate that two gene ranking lists do not correlate with each other due to a small number of samples and different feature selection mechanisms. However, the gene with a higher ranking in the merged gene ranking list represents its high relevance in both models, so false positive of relevant genes is limited. We then focused on protein function of top 50 genes from the merged gene ranking list.

**Figure 5:**
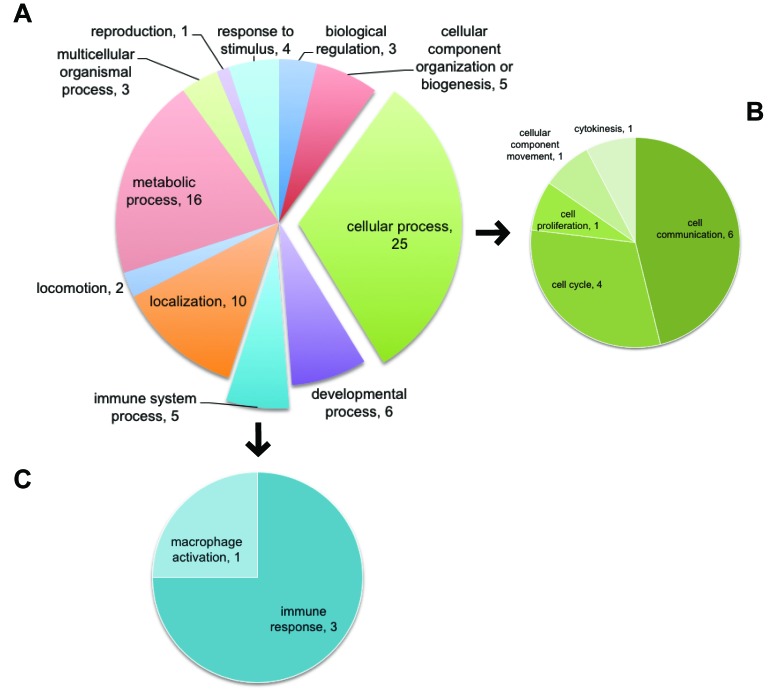
Using top 50 genes selected by Machine Learning, PANTHER identified GOs of cell cycle and cell proliferation affected by L2 expression Top 50 genes of combined SVM and RF scores were used as input for PANTHER. Biological processes were analyzed. **A.** All affected GOs. Numbers in each scallop indicate the number of genes identified in the 50 genes list that are classified as members of the corresponding GO. Explored scallops representing cellular process and immune system process were further illustrated in **B.** and **C.** B: GOs categorized as part of the cellular process. C: GOs categorized as part of the immune system process.

To gain a further understanding of the top 50 relevant genes, we focused on their biological functional interpretation. Interesting information was gathered about different biological processes and pathways that could be affected by 16L2 expression. Top 50 relevant genes were analyzed in PANTHER (Protein ANalysis THrough Evolutionary Relationships, http://pantherdb.org) [26], 11 categories of in total 84 biological processes affected upon 16L2 expression were uncovered by PANTHER. Genes in our dataset that were identified by PANTHER as hits in GOs were listed in Tables 3 and 4. As shown in both Figure 5 and Table 4, within the largest GOs hit by PANTHER, cell cycle and cell proliferation were highlighted, and five genes were identified as contributors for the changes in these GOs. In Figure 5, we highlighted the sub-categorical processes within Cellular Process (GO:0009987) in panel B. As listed in Table 4, five genes were related to cell cycle (GO: 0007049) and cell proliferation (GO: 00082083), FGF13, S100A7, ACTBL2, PARD6B, and NGFR. See also Supplementary Table 6 for the list of all 50 genes used here.

**Table 3:** PANTHER identified genes altered by L2.

GOs	Mapped Gene IDs
Biological Regulation (GO:0065007)	NGFR, SERPINB3, RAB26
Cellular Component Organization or Biogenesis (GO:0071840)	NGFR, SPTA1, RAB26, ACTBL2, SNX10
Cellular Process (GO:0009987)	ACTBL2, SIRPB2, TTC9, SNX10, LCN2, KLHDC7B, LGALSL, SSC4D, CLR1, ABCA5, ABCA4, NGFR, FA2H, NEURL3, SPTA1, LCE5A, PTPLAD2, SIRPB2, ARL14, RAB26, FGF13, S100A7, CAMK4
Developmental Process (GO:0032502)	ZNF608, NGFR, NEURL3, LCE5A, FGF13, CD274
Immune System Process (GO:0002376)	OLR1, NGFR, HSPB3, S100A7, C3
Localization (GO:0051179)	SSC4D, ABCA5, ABCA4, ARL14, RAB26, SLC7A11, ACTBL2, LCN2, PARD6B, SNX10
Locomotion (GO:0040011)	NGFR, SEMA4A
Metabolic Process (GO:0008152)	SSC4D, APOLD1, DHRS9, ABCA5, FA2H, HSPB3, ABCA4, LCE5A, S100A7, CAMK4, PTPRG, ZFP57, TTC9, LCN2, KLHDC7B, C3
Multicellular Organismal Process (GO:0032501)	NGFR, LCE5A, RAB26
Reproduction (GO:0000003)	NEURL3
Response to Stimulus (GO:0050896)	NGFR, SEMA4A, HSPB3, CAMK4

**Table 4:** PANTHER results sub-categories.

GOs		Mapped Gene IDs
Cellular Process (GO:0009987)	Cell Communication (GO:0007154)	NGFR, PTPLAD2, RAB26, FGF13, CAMK4, PARD6B
	Cell Cycle (GO:0007049)	FGF13, S100A7, ACTBL2, PARD6B
	Cell Proliferation (GO:0008283)	NGFR
	Cellular Component Movement (GO:0006928)	NGFR
	Cytokinesis (GO:0000910)	ACTBL2
Immune System Process (GO:0002376)	Immune Response (GO:0006955)	OLR1, NGFR, C3
	Macrophage Activation (GO:0042116)	S100A7

We also listed genes related to immune response in both Figure 5C and Table 4. Four genes characterized in Immune System Process (GO:0002376) as PANTHER hits were OLR1, NGFR, C3, and S100A7. In fact, five more genes, CAMK4, LCN2, SEMA4A, CD274, and SIRPB2, in our list of 50 genes used in PANTHER analysis also participate in immunity and inflammation.

## DISCUSSION

In nature, papillomaviruses (PVs) take advantage of cellular machinery and pathways to accomplish viral production and the release of mature virions. The biology is such that PVs require a dividing non-differentiated cell for genome amplification. This requirement is a result of lack of DNA replication machinery in the viral genome, thus the dependency on cellular factors. The importance of L2 in various events of HPV 16 has been described. In order to expand on the quest to identify roles of L2, we attempted to generate HaCaTs expressing L2 protein. Although L2 could be expressed in HaCaTs, the cells appeared to change in phenotype or enter apoptosis. Here, by transfecting HPV16 L2 gene into HaCaTs, we demonstrated a shift of cell cycle phase distribution and detected the expression profile of key regulators of cell proliferation. We report that using RNA-seq technology we identified 2586 human genes differentially expressed upon expression of L2 protein. This is the first time that RNA-seq is incorporated in the study of effects of L2 on a human cell line.

As the regulator of G2-M transition of mitosis, Cdc2/Cyclin-B complex has been reported to be sufficient for mammalian cell to go through cell cycle for its compensation function of all three G1 Cdks (Cdk2, 4, and 6) [27]. Furthermore, it has been observed that Cdc2 participates in p53-independent abrogation of postmitotic checkpoint (also known as spindle checkpoint) induced by HPV16 oncoprotein E6 [28]. In early G1 phase, Rb is hypophosphorylated and binds to transcription factor E2F. As the progression of G1 and Cdk4/6 promoting cell cycle to pass G1 restriction point, hyperphosphorylation of Rb results in the release of E2F, which then enters nucleus and mediates S-phase gene transcription. It is as well evident that E2F1 can regulate Cdc2 expression [29]. High concentration of hypophosphorylated Rb is also one feature of postmitotic checkpoint [30]. To date, researches on Cdc2 and Rb functioning in HPV biology are limited to HPV oncoproteins, such as 16 E6, and E1^E4. Our data of L2 transfected HaCaT cell cycle phase distribution demonstrated a shift from G1 to S and G2/M phases. This result is compatible with our result of decreased Cdc2 abundance in HPV 16 L2 expressed cells. In fact, a wide range of viruses has the ability to induce G2/M arrest [31]. It was previously reported that human immunodeficiency virus (HIV) induces G2 arrest in infected cells and hence up-regulates viral production [32], and it was also evident that HPV16 E1^E4-induced cultured Saos-2 cells G2 arrest is associated with cytoplasmic retention of active Cdc2/Cyclin B1 complexes [33]. Given that E1^E4 expression occurs at the same time with L2 expression, and E1^E4 is requited for the onset of viral DNA amplification, J. Doorbar *et al.* hypothesized that G2 phase arrest may create an optimized intracellular environment for viral DNA replication and other late events [33]. If this is the case, it is quite possible that L2 can also facilitate generation of this required environment.

Decreased level of both total and Ser807 phosphorylated Rb protein were detected. We observed a dosage effect of L2 upon Cdc2 and Rb expression profile, as well (data not shown). Different amounts (62.5ng, 125ng, 500ng, 1μg, 2μg, and 3μg) of p16L2h plasmids were transfected into cultured HaCaT cells, and protein lysates were then harvested at 12h, 18h, 24h, and 48h post transfection, and phosphorylated Cdc2 and Rb levels were checked. While being consistent with results shown in Figure 2A, the effect of L2 on both pCdc2 and pRb (Ser807) developed faster with more plasmid used in transfection, i.e., when transfecting with more plasmid DNA, the changes appeared earlier as compared to transfection with less plasmid DNA. Phospho-Chk2 (Thr68), phospho-Rb (Ser795), phosphor-Chk1 (Ser15), and phosphor-p53 (Ser15) were also tested via Western Blotting, and none of them showed noticeable changes of levels (data not shown).

More interestingly, it is known that in the genome amplification stage of HPV life cycle, E7 can stimulate infected cells to re-enter into mitotic S-phase [34-37]. This effect is mainly approached through E7-Rb interaction. E7 can bind to Rb and displaces previously Rb-bind E2F, which in turn gets into the nucleus and participates in transcription of genes required for cell cycle to progress. However, once infected cells migrate into upper epithelial layers, E6 and E7 abundances are at very low level, whereas L2 expression gets started. Viral synthesis is still going on in these cells. To guarantee the amplification of viral DNA, L2 may now maintain the S-phase re-entry stimulated by E7 and E6, given the fact that L2 can decrease Rb level. Therefore we hypothesize that L2 may partly compensate E7 function in regulating host cell cycle through impacting active Rb abundance. Using HPV31 in an organotypic culture, Holmgren and colleagues suggested that L2 might be involved in the final stages of viral production by presenting data that L2 mutant systems produced less DNase resistant viral particles [8]. This lack of viral DNA encapsidation may be related to the loss of transcription of a cellular protein necessary for viral packaging. Further work needs to be done to address these phenomena.

This is the first time that RNA-seq is used for a large-scale screen of responding genes in human cells upon HPV16 L2 expression. We used plasmid DNA transfection to introduce L2 protein into HaCaTs because we believe that L2 has a role in the viral production. In current analysis of our RNA-seq data, an emphasis has been put on regulatory gene sets of cell proliferation and apoptosis. However, our data provides much more information in understanding functions of L2 protein. By focusing on L2 transfection, our results can serve as a valuable database for future works focusing on L2 protein function, and may, therefore, simplify studies of overall L2 functions. Also, GSEA is a strong tool for generating promising hypotheses, which can then be used to reveal more roles played by L2 in HPV life cycle, as well as its influences on host cell biological processes. It has already been shown that L2 can interact with host cell transcription factors TBX2 and TBX3 [38], we are continuing to explore that L2 itself can act as a transcription factor.

We chose Cdk6, TFGβ2, MAPK1, FAK, and Pyk2 to conduct the validation of our RNA-seq results on their mRNA levels, as well as their biological functions. Cdk6 interacts with D-type cyclins during G1 phase to form a complex with Rb, mediates the phosphorylation of Rb, and therefore facilitates the entrance into the cell cycle [39]. TFGβ2 is a member of the transforming growth factor family cytokines, which are ligands for TGF-beta receptors. Binding of TGFβ2 to its receptor leads to recruitment and activation of SMAD family transcription factors that regulate gene expression [40, 41]. Many cellular processes can be regulated through TGF-SMAD pathway, including proliferation, differentiation, adhesion, migration, and other functions [42]. Increases of TGFβ2 mRNA and protein level in our results indicate an activation of TGF mediated signaling. Alteration of expression of several other downstream factors of TGF-β receptors, such as Smad3, MAPK1, and Jnk, were also demonstrated by our RNA-seq results. Further studies to pursue the mechanism of how TGF-β signaling pathways are utilized by HPV16 to accomplish its life cycle is worth great attention. MAPK1, also called p42 or ERK2, is as well involved in proliferation. Active MAPK1 can translocate to the nucleus and phosphorylate its nuclear targets [43]. To date, about 200 distinct targets for MAPKs have been identified[44,45]. Many of these target genes are involved the regulation of transcription and initiation of mitosis [46-48]. MAPK1 also has a transcriptional repressor function that is independent of its kinase activity [49]. Focal adhesion kinase (FAK, also known as protein tyrosine kinase 2, PTK2) and Pyk2 (protein tyrosine kinase 2 beta) are members of FAK subfamily that regulate reorganization of the actin cytoskeleton, adhesion, cell migration, cell polarization, and spreading. FAK subfamily, as a bridge between cytoskeletal and ERK signaling, promotes the activation of MAP kinase signaling cascade, including activation of MAPK1/ERK2, MAPK3/ERK1 and MAPK8/JNK1, and cellular response to TGF-β [50-52]. Our lab has previously reported that knock down of Pyk2 causes infectious HPV16 PsVs retention in trans-Golgi network, thus leads to a decrease of infection [53]. In this study, we identified an approximate 50% increase in Pyk2 protein level upon 16L2 expression, whereas no change of Pyk2 mRNA level observed. This result suggests that L2 may mediate the stabilization of Pyk2 protein without altering its mRNA expression.

In summary, genome-wide mRNA profiling in the presence of HPV16 L2 in HaCaTs identified a large amount of genes (2586) that uniquely respond to L2 expression. These genes covered more than 300 gene sets, revealing a great manipulation of cell biological pathways by L2. These findings provide a database that is extremely valuable for L2 function studies and selection of research targets and directions. Our data showing a shift of L2 positive cells toward mitotic S-phase, together with Cdc2 and Rb expression changes, provide evidence and support that L2 has an impact on host cell cycle progression. This leads to the hypothesis that L2 may, to some degree, compensate for E7 function in stimulating host cell division and may contribute to the homeostasis of the cell during initial entry and most importantly during viral production.

## MATERIALS AND METHODS

### Cell culture, plasmids, and antibodies

HaCaT cells (in vitro spontaneously transformed keratinocytes from histologically normal skin [54]) were purchased from AddexBio (San Diego, CA). Cells were cultured in Dulbecco’s Modified Eagle’s media (DMEM) supplemented with 10% fetal bovine serum (FBS). Control empty vector pA3M (pcDNA3 that encodes three copies of Myc epitope) was a gift from Dr. Robertson (Univ. of Pennsylvania School of Medicine, Philadelphia, PA) [55]. All other plasmids (p8fwb, p16L1h, and p16L2h) were derived from pA3M with addition of coding region of eGFP, HPV16 L1h (16L1h), and HPV16 L2h (16L2h), respectively, and were obtained from Dr. Schiller (NCI, Baltimore MD) [56]. The letter “h” represents the codon optimization of the coding regions of 16L1h and 16L2h without changing the amino acid sequence. Mouse monoclonal antibody (mAb) POH1 (anti-Cdc2), rabbit mAb D20 (anti-Rb), rabbit mAb against phospho-Rb (Ser807/811) D20B12, and rabbit polyclonal phospho-Cdc2 (Tyr15) antibody (product # 9111) purchased from Cell Signaling Technology (Danvers, MA).

### Transfection

HaCaT cells were seeded into 6-well plate (3-4 x 10^5^ cells/well) and cultured overnight. Fresh media was changed 30 minutes prior to transfection. GenJet™ DNA in vitro transfection reagent (SignaGen Laboratories, Cat#SL10048) was used as GenJet™: DNA ratio (volume: mass) of 3:1. Cells were transfected when approximately 60% confluence. Transfection was done following manufacture’s instruction and cells were cultured for 18h before sample collection. Transfection efficiency was examined by parallel eGFP plasmid (8fwb) transfection under identical conditions. The 8fwb plasmid is of similar size of p16L1h and p16L2h. Transfection efficiency was checked by fluorescence microscope and flow cytometer.

### DNA content detection with Propidium Iodide (PI)

1.0-1.2 x 10^6^ cells were collected 18h post transfection. After trypsinization cells were washed three times with cold PBS and fixed overnight in 80% Ethanol at -20°C. Cells were collected by centrifugation next day and rehydrated in PBS for 10min. Three washes with PBS were used to remove potentially remaining Ethanol. Cells were then suspended in 500μl Propidium Iodide (PI) staining solution (PBS solution with 1% Triton X-100, 20μg/ml PI, and 0.2mg/ml RNase A). DNA content detection was performed with BD Accuri™ C6 flow cytometer (BD Biosciences, San Jose, CA) and FL2-A was used for indication of DNA content.

### RNA extraction and reverse transcription

Total cellular RNA was extracted from cells using RNeasy minikit (Cat#: 74104, Qiagen, Alameda, CA). RNA was reverse transcribed to cDNA using QIAGEN LongRange 2Step RT-PCR Kit using oligo-dT primer as per manufacture’s instruction (Cat#: 205922, Qiagen).

### Real-time PCR and primers

Real-time PCR (qPCR) starting with cDNAs was performed using QuantiTect SYBR Green PCR Kit (Cat#: 204143, Qiagen). qPCR was run on an Applied Biosystems^®^ 7500 Real-Time PCR System (Applied Biosystems, Waltham, MA) in triplicate per each sample and gene. PCR primers used: Rb forward primer: 5’- TTGGATCACAGCGATACAAACTT -3’; Rb reverse primer: 5’- AGCGCACGCCAATAAAGACAT -3’; Cdc2 forward primer: 5’-CAGACTAGAAAGTGAAGAGGAAGG -3’; Cdc2 reverse primer: 5’- AAGAATCCATGTACTGACCAGG -3’; β-Actin forward primer: 5’- CTGGAACGGTGAAGGTGACA -3’; β-Actin reverse primer: 5’- AAGGGACTTCCTGTAACAATGCA -3’. β-Actin was used as a control for RNA loading and reverse real-time PCR efficiency.

### RNA sequencing

15μg cellular RNA was used for RNA quality assess (BioAnalyzer from Agilent Technologies) and mRNA library preparation. The mRNAs were fragmented, and the first strand of cDNA was synthesized from the cleaved RNA using random primers followed by second strand cDNA synthesis. The purified cDNA templates were enriched by PCR amplification to generate cDNA libraries. The cDNA libraries were presented to RNA sequencing facility, and two rapid single-read 50 Illumina HiSeq sequencing runs were performed. Raw reads from separate lanes of the same sample were merged before mapping. RNA sequencing was performed at New York University Langone Medical Center Genome Technology Science Laboratory (New York, NY).

### Bioinformatics and biostatistics analysis

#### Reads mapping and differential gene expression analysis (DGE)

Raw reads of sequencing were mapped with Bowtie1 (version 1.0.0), which was widely used for short reads (no more than 50 bp) mapping, with two mismatches allowed. Reads normalization was done with the function for estimation of size factor in DESEQ2 as descriped before [57]. The unique mapped reads were reported and subjected to subsequent necessary processing and PCR duplicates removal before assigning to gene model (hg19, ignome version). Standard principal component analysis (PCA) implementation in R (prcomp function) was used, which is stated by R: https://stat.ethz.ch/R-manual/R-patched/library/stats/html/prcomp.html. For DGE statistical analysis, DESeq2 R/Bioconductor package in the R statistical programming environment was used. The p-value for DEG was determined by unpaired two-tailed t-test with unequal variance and it is adjusted by Fisher-Yates methods. The false discovery rate (q-value) was calculated for each gene using Storey and Tibshirani methods [58].

#### Relevant genes prediction through machine learning

RNA-seq data was log2 transformed, and 8fwb and 16L2h groups were used as two classifications. To obtain candidate 16L2h relevant genes, we used Support Vector Machine (SVM) and Random Forest (RF). These two machine learning algorithms worked for sample classification based on different complex patterns recognition. They were both commonly used for gene selection [59-63]. Random Forest was performed in MATLAB package (MATLAB and Statistics Toolbox Release 2016a, The MathWorks, Inc., Natick, Massachusetts, United States). SVM feature selection was performed by WEKA package [64, 65].

SVM is a classic algorithm for classification. In this study, we used linear SVM for the feature selection, though SVM could be customized by other kernel methods and generated to handle non-linear boundaries when the dataset size was very large. Also, SVM is a commonly used method for gene selection based on recursive feature elimination [59]. In this model, sign function was used to convert numerical output from input genes expression to categorical label. The model was defined by the following equation:

$$Y\left(g\right)=sgn\left(w^{T}g+b\right)$$ (1)

Where Y was classifications of samples, w=[w_1_, w_2_, … ,w_n_]^T^ was the weight vector for genes and g=[g_1_, g_2_, … , g_n_]^T^ was a vector of gene expression, n was the number of genes in this model. The gene with a large absolute value of weight represents strong importance for the classification of samples. Recursive feature elimination means that irrelevant genes are eliminated step by step, and the SVM was retrained with less and less number of genes. In each round, the gene with smallest absolute weight was removed. The whole process was recursive until it achieved good classification performance.

RF is an ensemble of decision trees developed by Leo Breiman [66]. Each decision tree was calculated from a bootstrap sample of the training data with a subset of features that were called split nodes. The split nodes of the tree were from a subset of genes which were randomly selected from the whole set of genes. At the same time, the importance of features could be measured through Out-of-Bag error rate which was related with classification accuracy [67]. In this study, we built the random forest with 500 decision trees.

Since two models were used in this study, we combined these gene lists by Ensemble Feature Selection to reduce bias and increase the credibility of selected genes [68,69]. Each model generated a unique gene relevance list and the important genes were assigned to higher ranks in the list. Two lists were combined through rank combination. Genes with high rankings indicated they very likely played important roles in the process of cell biology with p16L2h transfection.

#### Gene set enrichment analysis and leading edge analysis

GSEA v2.0.6 was used for this analysis (Broad Institute, Cambridge, MA, USA) [20, 21]. A pairwise comparison between L2h and 8fwb transfection group was performed using ranked gene expression profiles from DGE analysis. To be specific, all significant DEGs in the comparison between L2h and 8fwb (Supplementary Table 2, sheet 3) were ranked based on their p-values and adjusted p-values in DGE result. LogFC and number of each gene calculated by DGE analysis were used as input for GSEA. Ranked genes were mapped to C5: GO gene sets in MSigDB, which contains 1454 gene sets. Gene sets in this collection are derived from the controlled vocabulary of the Gene Ontology (GO) project: The Gene Ontology Consortium. The gene sets are based on GO terms (gene_ontology_edit.obo, downloaded 1/25/2008) and their associations to human genes (gene2go, downloaded 1/22/2008). The enrichment scores were calculated by walking down the ordered list.

## SUPPLEMENTARY MATERIALS FIGURES AND TABLES















## Abbreviations

16L2: HPV16 L2
DGE: differential gene expression analysis
DEG: differentially expressed gene
PCA: principle component analysis
SVM: support vector machine
RF: random forest
GSEA: gene set enrichment analysis
LEA: leading edge analysis
