# Supplementary material for: Host cell transcriptome modification upon exogenous HPV16 L2 protein expression
Source: Oncotarget. 2017 Oct 12;8(53):90730–47. doi: 10.18632/oncotarget.21817 (PMC5710881; doi:10.18632/oncotarget.21817)
Supplement: Supplementary file 1 [file oncotarget-08-90730-s001.pdf]

Host cell transcriptome modification upon exogenous HPV16 L2 protein expression

SUPPLEMENTARY MATERIALS

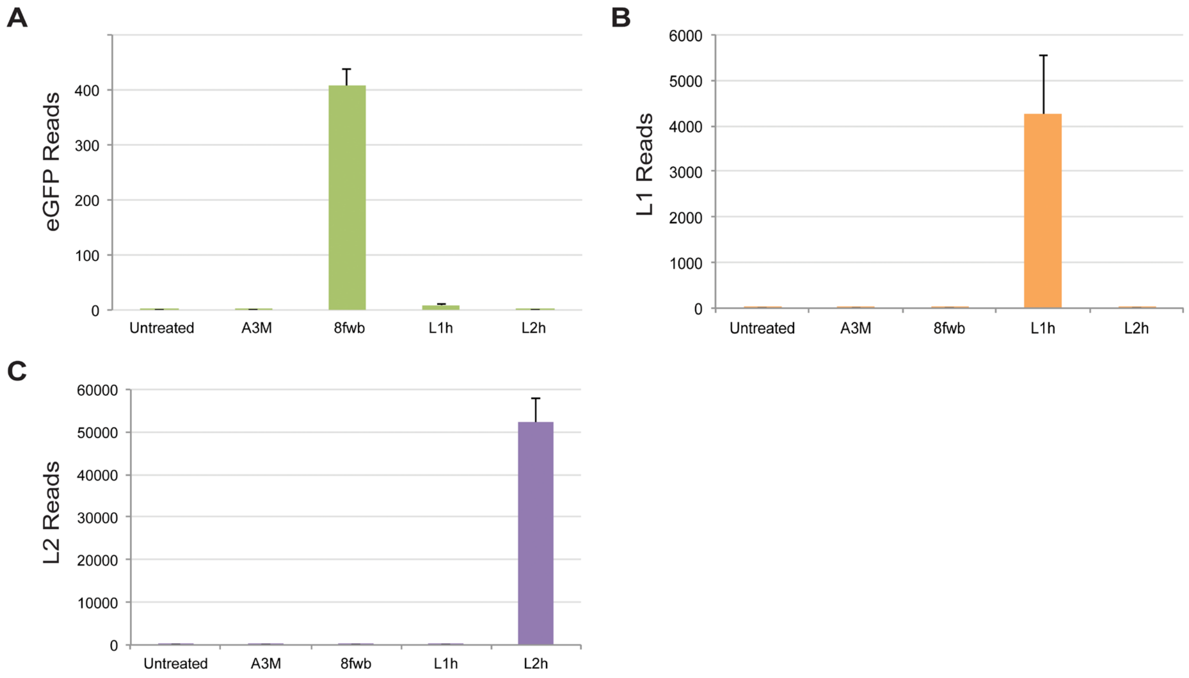

Supplementary Figure 1: Pseudo-genome mapping for RNA-Seq reads. DNA coding sequences of eGFP, L1h, and L2h genes embedded in transfected plasmids were used to generate pseudo-genomes of the same genes. RNA-Seq reads are then mapped back to pseudo-genomes for exogenous gene expression check. Average numbers of reads and standard deviations are indicated above in graphs.

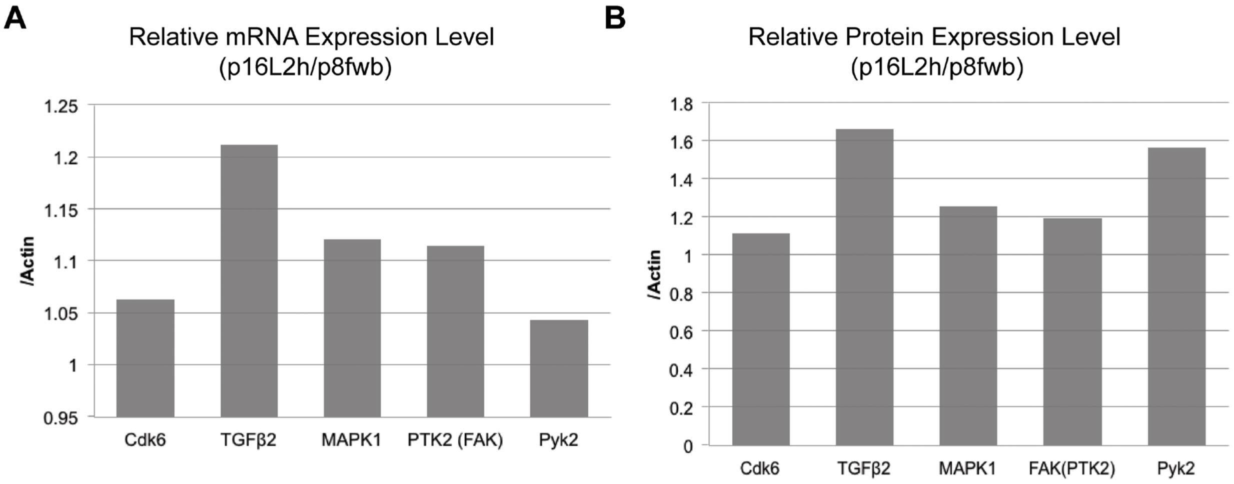

Supplementary Figure 2. Quantification of mRNA and protein levels of five genes confirmed RNA-seq results. HaCaTs were transfected with either p8fwb or p16L2h plasmid DNA, and cells were harvested for RNA or total protein preparation. Experiments were repeated three times (with 2.5μg plasmid). For qRT-PCR, standard curve method was used to generate data, and an average number of three repeats were first normalized to Actin mRNA quantity, then normalized to p8fwb transfected group. Same normalization was done with Western Blotting results, and the relative expression levels were plotted.

For Supplementary Tables see in Supplementary Files

Supplementary Table 1. RNA-seq results after alignment. Sheet 1: Log2 transformed counts for each sequencing sample. Sheet 2: Sample alignment statistics.

Supplementary Table 2. Differential Gene Expression (DGE) analysis results. Different comparisons are listed in sheets.

Supplementary Table 3. Unique genes in Fig 2E and 2F, the Venn Diagrams.

Supplementary Table 4. Gene Set Enrichment Analysis for positively regulated gene sets in L2h_8fwb comparison.

Supplementary Table 5. Gene Set Enrichment Analysis for negatively regulated gene sets in L2h_8fwb comparison.

Supplementary Table 6. Top 50 genes in combined ranking list of Support Vector Machine and Random Forest.
