# Supplementary material for: Host cell transcriptome modification upon exogenous HPV16 L2 protein expression
Source: Oncotarget. 2017 Oct 12;8(53):90730–47. doi: 10.18632/oncotarget.21817 (PMC5710881; doi:10.18632/oncotarget.21817)
Supplement: Supplementary file 5 [file oncotarget-08-90730-s005.docx]

**Table S4. Gene Set Enrichment Analysis for positively regulated gene sets in L2h_8fwb comparison.**

|  | **Gene Sets**  **follow link to MSigDB** | **SIZE** | **NOM**  **p-val** | **FDR**  **q-val** | **RANK AT MAX** | **LEADING EDGE** |
| --- | --- | --- | --- | --- | --- | --- |
| 1 | [MULTI_ORGANISM_PROCESS](http://www.broadinstitute.org/gsea/msigdb/cards/MULTI_ORGANISM_PROCESS) | 34 | 0.000 | 0.000 | 944 | tags=79%, list=30%, signal=113% |
| 2 | [POSITIVE_REGULATION_OF_BIOLOGICAL_PROCESS](http://www.broadinstitute.org/gsea/msigdb/cards/POSITIVE_REGULATION_OF_BIOLOGICAL_PROCESS) | 146 | 0.000 | 0.000 | 2002 | tags=85%, list=65%, signal=228% |
| 3 | [POSITIVE_REGULATION_OF_CELLULAR_PROCESS](http://www.broadinstitute.org/gsea/msigdb/cards/POSITIVE_REGULATION_OF_CELLULAR_PROCESS) | 140 | 0.000 | 0.000 | 2002 | tags=85%, list=65%, signal=229% |
| 4 | [NUCLEUS](http://www.broadinstitute.org/gsea/msigdb/cards/NUCLEUS) | 295 | 0.000 | 0.000 | 2137 | tags=82%, list=69%, signal=239% |
| 5 | [DEFENSE_RESPONSE](http://www.broadinstitute.org/gsea/msigdb/cards/DEFENSE_RESPONSE) | 45 | 0.000 | 0.000 | 1412 | tags=80%, list=46%, signal=145% |
| 6 | [NEGATIVE_REGULATION_OF_CELLULAR_PROCESS](http://www.broadinstitute.org/gsea/msigdb/cards/NEGATIVE_REGULATION_OF_CELLULAR_PROCESS) | 139 | 0.000 | 0.000 | 1965 | tags=83%, list=63%, signal=215% |
| 7 | [NEGATIVE_REGULATION_OF_BIOLOGICAL_PROCESS](http://www.broadinstitute.org/gsea/msigdb/cards/NEGATIVE_REGULATION_OF_BIOLOGICAL_PROCESS) | 147 | 0.000 | 0.000 | 1965 | tags=82%, list=63%, signal=214% |
| 8 | [IMMUNE_RESPONSE](http://www.broadinstitute.org/gsea/msigdb/cards/IMMUNE_RESPONSE) | 32 | 0.000 | 0.002 | 558 | tags=56%, list=18%, signal=68% |
| 9 | [TRANSCRIPTION](http://www.broadinstitute.org/gsea/msigdb/cards/TRANSCRIPTION) | 166 | 0.000 | 0.002 | 1979 | tags=80%, list=64%, signal=209% |
| 10 | [BIOPOLYMER_METABOLIC_PROCESS](http://www.broadinstitute.org/gsea/msigdb/cards/BIOPOLYMER_METABOLIC_PROCESS) | 340 | 0.000 | 0.003 | 1969 | tags=74%, list=63%, signal=181% |
| 11 | [SIGNAL_TRANSDUCTION](http://www.broadinstitute.org/gsea/msigdb/cards/SIGNAL_TRANSDUCTION) | 299 | 0.000 | 0.003 | 1621 | tags=64%, list=52%, signal=121% |
| 12 | [RNA_METABOLIC_PROCESS](http://www.broadinstitute.org/gsea/msigdb/cards/RNA_METABOLIC_PROCESS) | 161 | 0.000 | 0.004 | 1699 | tags=71%, list=55%, signal=148% |
| 13 | [IMMUNE_SYSTEM_PROCESS](http://www.broadinstitute.org/gsea/msigdb/cards/IMMUNE_SYSTEM_PROCESS) | 49 | 0.000 | 0.004 | 558 | tags=47%, list=18%, signal=56% |
| 14 | [REGULATION_OF_DEVELOPMENTAL_PROCESS](http://www.broadinstitute.org/gsea/msigdb/cards/REGULATION_OF_DEVELOPMENTAL_PROCESS) | 101 | 0.000 | 0.007 | 1965 | tags=83%, list=63%, signal=219% |
| 15 | [REGULATION_OF_METABOLIC_PROCESS](http://www.broadinstitute.org/gsea/msigdb/cards/REGULATION_OF_METABOLIC_PROCESS) | 176 | 0.000 | 0.010 | 2135 | tags=83%, list=69%, signal=251% |
| 17 | [RESPONSE_TO_OTHER_ORGANISM](http://www.broadinstitute.org/gsea/msigdb/cards/RESPONSE_TO_OTHER_ORGANISM) | 17 | 0.000 | 0.011 | 1098 | tags=82%, list=35%, signal=127% |
| 20 | [POSITIVE_REGULATION_OF_CELLULAR_METABOLIC_PROCESS](http://www.broadinstitute.org/gsea/msigdb/cards/POSITIVE_REGULATION_OF_CELLULAR_METABOLIC_PROCESS) | 48 | 0.000 | 0.011 | 2002 | tags=92%, list=65%, signal=254% |
| 22 | [REGULATION_OF_CELLULAR_METABOLIC_PROCESS](http://www.broadinstitute.org/gsea/msigdb/cards/REGULATION_OF_CELLULAR_METABOLIC_PROCESS) | 175 | 0.000 | 0.012 | 2058 | tags=81%, list=66%, signal=226% |
| 26 | [RESPONSE_TO_EXTERNAL_STIMULUS](http://www.broadinstitute.org/gsea/msigdb/cards/RESPONSE_TO_EXTERNAL_STIMULUS) | 61 | 0.000 | 0.016 | 759 | tags=48%, list=24%, signal=62% |
| 29 | [BEHAVIOR](http://www.broadinstitute.org/gsea/msigdb/cards/BEHAVIOR) | 21 | 0.000 | 0.020 | 759 | tags=62%, list=24%, signal=81% |
| 33 | [NUCLEAR_PART](http://www.broadinstitute.org/gsea/msigdb/cards/NUCLEAR_PART) | 93 | 0.000 | 0.024 | 2156 | tags=87%, list=69%, signal=277% |
| 16 | [CYTOKINE_ACTIVITY](http://www.broadinstitute.org/gsea/msigdb/cards/CYTOKINE_ACTIVITY) | 22 | 0.002 | 0.011 | 168 | tags=45%, list=5%, signal=48% |
| 18 | [NEGATIVE_REGULATION_OF_DEVELOPMENTAL_PROCESS](http://www.broadinstitute.org/gsea/msigdb/cards/NEGATIVE_REGULATION_OF_DEVELOPMENTAL_PROCESS) | 40 | 0.002 | 0.010 | 1845 | tags=90%, list=59%, signal=219% |
| 23 | [REPRODUCTIVE_PROCESS](http://www.broadinstitute.org/gsea/msigdb/cards/REPRODUCTIVE_PROCESS) | 29 | 0.002 | 0.011 | 987 | tags=66%, list=32%, signal=95% |
| 25 | [ECTODERM_DEVELOPMENT](http://www.broadinstitute.org/gsea/msigdb/cards/ECTODERM_DEVELOPMENT) | 28 | 0.002 | 0.015 | 932 | tags=64%, list=30%, signal=91% |
| 28 | [REGULATION_OF_GENE_EXPRESSION](http://www.broadinstitute.org/gsea/msigdb/cards/REGULATION_OF_GENE_EXPRESSION) | 152 | 0.002 | 0.017 | 1979 | tags=78%, list=64%, signal=206% |
| 43 | [REPRODUCTION](http://www.broadinstitute.org/gsea/msigdb/cards/REPRODUCTION) | 37 | 0.002 | 0.030 | 1023 | tags=59%, list=33%, signal=88% |
| 45 | [REGULATION_OF_APOPTOSIS](http://www.broadinstitute.org/gsea/msigdb/cards/REGULATION_OF_APOPTOSIS) | 77 | 0.002 | 0.030 | 1998 | tags=83%, list=64%, signal=228% |
| 19 | [EXTRACELLULAR_REGION](http://www.broadinstitute.org/gsea/msigdb/cards/EXTRACELLULAR_REGION) | 82 | 0.004 | 0.010 | 624 | tags=41%, list=20%, signal=51% |
| 24 | [RNA_BIOSYNTHETIC_PROCESS](http://www.broadinstitute.org/gsea/msigdb/cards/RNA_BIOSYNTHETIC_PROCESS) | 144 | 0.004 | 0.016 | 1913 | tags=77%, list=62%, signal=192% |
| 27 | [NUCLEOBASENUCLEOSIDENUCLEOTIDE_AND_NUCLEIC_ACID_METABOLIC_PROCESS](http://www.broadinstitute.org/gsea/msigdb/cards/NUCLEOBASENUCLEOSIDENUCLEOTIDE_AND_NUCLEIC_ACID_METABOLIC_PROCESS) | 244 | 0.004 | 0.016 | 2002 | tags=76%, list=65%, signal=197% |
| 30 | [REGULATION_OF_CELLULAR_PROTEIN_METABOLIC_PROCESS](http://www.broadinstitute.org/gsea/msigdb/cards/REGULATION_OF_CELLULAR_PROTEIN_METABOLIC_PROCESS) | 26 | 0.004 | 0.022 | 1340 | tags=77%, list=43%, signal=134% |
| 32 | [POSITIVE_REGULATION_OF_METABOLIC_PROCESS](http://www.broadinstitute.org/gsea/msigdb/cards/POSITIVE_REGULATION_OF_METABOLIC_PROCESS) | 49 | 0.004 | 0.021 | 2002 | tags=90%, list=65%, signal=249% |
| 34 | [INTRACELLULAR_SIGNALING_CASCADE](http://www.broadinstitute.org/gsea/msigdb/cards/INTRACELLULAR_SIGNALING_CASCADE) | 139 | 0.004 | 0.023 | 1603 | tags=66%, list=52%, signal=131% |
| 35 | [POSITIVE_REGULATION_OF_SIGNAL_TRANSDUCTION](http://www.broadinstitute.org/gsea/msigdb/cards/POSITIVE_REGULATION_OF_SIGNAL_TRANSDUCTION) | 31 | 0.004 | 0.023 | 1649 | tags=84%, list=53%, signal=177% |
| 36 | [REGULATION_OF_PROTEIN_METABOLIC_PROCESS](http://www.broadinstitute.org/gsea/msigdb/cards/REGULATION_OF_PROTEIN_METABOLIC_PROCESS) | 28 | 0.004 | 0.023 | 1340 | tags=75%, list=43%, signal=131% |
| 40 | [TRANSCRIPTION_FROM_RNA_POLYMERASE_II_PROMOTER](http://www.broadinstitute.org/gsea/msigdb/cards/TRANSCRIPTION_FROM_RNA_POLYMERASE_II_PROMOTER) | 94 | 0.004 | 0.024 | 1913 | tags=79%, list=62%, signal=199% |
| 42 | [NEGATIVE_REGULATION_OF_PROGRAMMED_CELL_DEATH](http://www.broadinstitute.org/gsea/msigdb/cards/NEGATIVE_REGULATION_OF_PROGRAMMED_CELL_DEATH) | 29 | 0.004 | 0.031 | 1845 | tags=90%, list=59%, signal=219% |
| 44 | [EPIDERMIS_DEVELOPMENT](http://www.broadinstitute.org/gsea/msigdb/cards/EPIDERMIS_DEVELOPMENT) | 26 | 0.004 | 0.030 | 932 | tags=62%, list=30%, signal=87% |
| 47 | [NEGATIVE_REGULATION_OF_CELLULAR_METABOLIC_PROCESS](http://www.broadinstitute.org/gsea/msigdb/cards/NEGATIVE_REGULATION_OF_CELLULAR_METABOLIC_PROCESS) | 63 | 0.004 | 0.034 | 2131 | tags=89%, list=69%, signal=278% |
| 21 | [TRANSCRIPTION_DNA_DEPENDENT](http://www.broadinstitute.org/gsea/msigdb/cards/TRANSCRIPTION_DNA_DEPENDENT) | 144 | 0.006 | 0.012 | 1913 | tags=77%, list=62%, signal=192% |
| 31 | [PROTEIN_KINASE_CASCADE](http://www.broadinstitute.org/gsea/msigdb/cards/PROTEIN_KINASE_CASCADE) | 79 | 0.006 | 0.022 | 1589 | tags=71%, list=51%, signal=142% |
| 38 | [RESPONSE_TO_WOUNDING](http://www.broadinstitute.org/gsea/msigdb/cards/RESPONSE_TO_WOUNDING) | 36 | 0.006 | 0.024 | 759 | tags=53%, list=24%, signal=69% |
| 57 | [CELL_CELL_SIGNALING](http://www.broadinstitute.org/gsea/msigdb/cards/CELL_CELL_SIGNALING) | 53 | 0.006 | 0.047 | 225 | tags=28%, list=7%, signal=30% |
| 39 | [CELL_DEVELOPMENT](http://www.broadinstitute.org/gsea/msigdb/cards/CELL_DEVELOPMENT) | 119 | 0.008 | 0.024 | 1845 | tags=75%, list=59%, signal=177% |
| 50 | [APOPTOSIS_GO](http://www.broadinstitute.org/gsea/msigdb/cards/APOPTOSIS_GO) | 97 | 0.008 | 0.040 | 1965 | tags=79%, list=63%, signal=210% |
| 48 | [TRANSCRIPTION_FACTOR_ACTIVITY](http://www.broadinstitute.org/gsea/msigdb/cards/TRANSCRIPTION_FACTOR_ACTIVITY) | 77 | 0.010 | 0.038 | 968 | tags=49%, list=31%, signal=70% |
| 49 | [NEGATIVE_REGULATION_OF_METABOLIC_PROCESS](http://www.broadinstitute.org/gsea/msigdb/cards/NEGATIVE_REGULATION_OF_METABOLIC_PROCESS) | 63 | 0.010 | 0.039 | 2131 | tags=89%, list=69%, signal=278% |
| 52 | [REGULATION_OF_TRANSCRIPTION](http://www.broadinstitute.org/gsea/msigdb/cards/REGULATION_OF_TRANSCRIPTION) | 136 | 0.010 | 0.041 | 1979 | tags=77%, list=64%, signal=204% |
| 53 | [PROGRAMMED_CELL_DEATH](http://www.broadinstitute.org/gsea/msigdb/cards/PROGRAMMED_CELL_DEATH) | 98 | 0.010 | 0.042 | 1965 | tags=80%, list=63%, signal=210% |
| 54 | [MEMBRANE_ENCLOSED_LUMEN](http://www.broadinstitute.org/gsea/msigdb/cards/MEMBRANE_ENCLOSED_LUMEN) | 69 | 0.010 | 0.042 | 2156 | tags=88%, list=69%, signal=283% |
| 46 | [NEGATIVE_REGULATION_OF_APOPTOSIS](http://www.broadinstitute.org/gsea/msigdb/cards/NEGATIVE_REGULATION_OF_APOPTOSIS) | 28 | 0.012 | 0.034 | 1845 | tags=89%, list=59%, signal=218% |
| 55 | [REGULATION_OF_SIGNAL_TRANSDUCTION](http://www.broadinstitute.org/gsea/msigdb/cards/REGULATION_OF_SIGNAL_TRANSDUCTION) | 53 | 0.012 | 0.044 | 2064 | tags=87%, list=67%, signal=255% |
| 65 | [ORGAN_DEVELOPMENT](http://www.broadinstitute.org/gsea/msigdb/cards/ORGAN_DEVELOPMENT) | 119 | 0.012 | 0.058 | 649 | tags=34%, list=21%, signal=42% |
| 37 | [ORGANELLE_ORGANIZATION_AND_BIOGENESIS](http://www.broadinstitute.org/gsea/msigdb/cards/ORGANELLE_ORGANIZATION_AND_BIOGENESIS) | 107 | 0.013 | 0.025 | 2053 | tags=82%, list=66%, signal=235% |
| 41 | [REGULATION_OF_PROGRAMMED_CELL_DEATH](http://www.broadinstitute.org/gsea/msigdb/cards/REGULATION_OF_PROGRAMMED_CELL_DEATH) | 78 | 0.013 | 0.024 | 1998 | tags=83%, list=64%, signal=228% |
| 56 | [REGULATION_OF_NUCLEOBASENUCLEOSIDENUCLEOTIDE_AND_NUCLEIC_ACID_METABOLIC_PROCESS](http://www.broadinstitute.org/gsea/msigdb/cards/REGULATION_OF_NUCLEOBASENUCLEOSIDENUCLEOTIDE_AND_NUCLEIC_ACID_METABOLIC_PROCESS) | 145 | 0.016 | 0.044 | 1979 | tags=77%, list=64%, signal=201% |
| 58 | [TISSUE_DEVELOPMENT](http://www.broadinstitute.org/gsea/msigdb/cards/TISSUE_DEVELOPMENT) | 38 | 0.016 | 0.047 | 1040 | tags=58%, list=34%, signal=86% |
| 61 | [POSITIVE_REGULATION_OF_DEVELOPMENTAL_PROCESS](http://www.broadinstitute.org/gsea/msigdb/cards/POSITIVE_REGULATION_OF_DEVELOPMENTAL_PROCESS) | 53 | 0.016 | 0.052 | 1998 | tags=85%, list=64%, signal=234% |
| 67 | [RESPONSE_TO_BIOTIC_STIMULUS](http://www.broadinstitute.org/gsea/msigdb/cards/RESPONSE_TO_BIOTIC_STIMULUS) | 27 | 0.016 | 0.062 | 946 | tags=59%, list=30%, signal=85% |
| 73 | [POSITIVE_REGULATION_OF_NUCLEOBASENUCLEOSIDENUCLEOTIDE_AND_NUCLEIC_ACID_METABOLIC_PROCESS](http://www.broadinstitute.org/gsea/msigdb/cards/POSITIVE_REGULATION_OF_NUCLEOBASENUCLEOSIDENUCLEOTIDE_AND_NUCLEIC_ACID_METABOLIC_PROCESS) | 35 | 0.016 | 0.067 | 2002 | tags=89%, list=65%, signal=247% |
| 51 | [ORGANELLE_LUMEN](http://www.broadinstitute.org/gsea/msigdb/cards/ORGANELLE_LUMEN) | 69 | 0.018 | 0.041 | 2156 | tags=88%, list=69%, signal=283% |
| 70 | [REGULATION_OF_I_KAPPAB_KINASE_NF_KAPPAB_CASCADE](http://www.broadinstitute.org/gsea/msigdb/cards/REGULATION_OF_I_KAPPAB_KINASE_NF_KAPPAB_CASCADE) | 22 | 0.018 | 0.062 | 1581 | tags=82%, list=51%, signal=166% |
| 68 | [RECEPTOR_BINDING](http://www.broadinstitute.org/gsea/msigdb/cards/RECEPTOR_BINDING) | 74 | 0.020 | 0.061 | 566 | tags=35%, list=18%, signal=42% |
| 69 | [TRANSCRIPTION_REPRESSOR_ACTIVITY](http://www.broadinstitute.org/gsea/msigdb/cards/TRANSCRIPTION_REPRESSOR_ACTIVITY) | 42 | 0.020 | 0.062 | 1583 | tags=74%, list=51%, signal=149% |
| 60 | [LOCOMOTORY_BEHAVIOR](http://www.broadinstitute.org/gsea/msigdb/cards/LOCOMOTORY_BEHAVIOR) | 17 | 0.022 | 0.051 | 1098 | tags=71%, list=35%, signal=109% |
| 64 | [NEGATIVE_REGULATION_OF_TRANSCRIPTION](http://www.broadinstitute.org/gsea/msigdb/cards/NEGATIVE_REGULATION_OF_TRANSCRIPTION) | 47 | 0.022 | 0.058 | 1965 | tags=85%, list=63%, signal=229% |
| 62 | [REGULATION_OF_TRANSCRIPTIONDNA_DEPENDENT](http://www.broadinstitute.org/gsea/msigdb/cards/REGULATION_OF_TRANSCRIPTIONDNA_DEPENDENT) | 117 | 0.024 | 0.056 | 1699 | tags=68%, list=55%, signal=145% |
| 63 | [EXTRACELLULAR_REGION_PART](http://www.broadinstitute.org/gsea/msigdb/cards/EXTRACELLULAR_REGION_PART) | 61 | 0.024 | 0.055 | 624 | tags=39%, list=20%, signal=48% |
| 84 | [TRANSCRIPTION_FACTOR_BINDING](http://www.broadinstitute.org/gsea/msigdb/cards/TRANSCRIPTION_FACTOR_BINDING) | 81 | 0.024 | 0.094 | 2135 | tags=84%, list=69%, signal=262% |
| 66 | [I_KAPPAB_KINASE_NF_KAPPAB_CASCADE](http://www.broadinstitute.org/gsea/msigdb/cards/I_KAPPAB_KINASE_NF_KAPPAB_CASCADE) | 28 | 0.025 | 0.060 | 2135 | tags=96%, list=69%, signal=306% |
| 59 | [REGULATION_OF_RNA_METABOLIC_PROCESS](http://www.broadinstitute.org/gsea/msigdb/cards/REGULATION_OF_RNA_METABOLIC_PROCESS) | 118 | 0.027 | 0.051 | 1699 | tags=69%, list=55%, signal=146% |
| 74 | [PROTEIN_METABOLIC_PROCESS](http://www.broadinstitute.org/gsea/msigdb/cards/PROTEIN_METABOLIC_PROCESS) | 244 | 0.027 | 0.069 | 2102 | tags=77%, list=68%, signal=219% |
| 71 | [POSITIVE_REGULATION_OF_CELL_PROLIFERATION](http://www.broadinstitute.org/gsea/msigdb/cards/POSITIVE_REGULATION_OF_CELL_PROLIFERATION) | 25 | 0.028 | 0.062 | 1482 | tags=76%, list=48%, signal=144% |
| 72 | [EXTRACELLULAR_SPACE](http://www.broadinstitute.org/gsea/msigdb/cards/EXTRACELLULAR_SPACE) | 44 | 0.028 | 0.063 | 740 | tags=45%, list=24%, signal=59% |
| 76 | [POSITIVE_REGULATION_OF_PROTEIN_METABOLIC_PROCESS](http://www.broadinstitute.org/gsea/msigdb/cards/POSITIVE_REGULATION_OF_PROTEIN_METABOLIC_PROCESS) | 15 | 0.031 | 0.071 | 1160 | tags=73%, list=37%, signal=117% |
| 80 | [POSITIVE_REGULATION_OF_TRANSCRIPTION](http://www.broadinstitute.org/gsea/msigdb/cards/POSITIVE_REGULATION_OF_TRANSCRIPTION) | 33 | 0.033 | 0.089 | 1701 | tags=79%, list=55%, signal=173% |
| 77 | [CYTOSKELETON_ORGANIZATION_AND_BIOGENESIS](http://www.broadinstitute.org/gsea/msigdb/cards/CYTOSKELETON_ORGANIZATION_AND_BIOGENESIS) | 60 | 0.034 | 0.077 | 2053 | tags=85%, list=66%, signal=246% |
| 75 | [REGULATION_OF_CELL_DIFFERENTIATION](http://www.broadinstitute.org/gsea/msigdb/cards/REGULATION_OF_CELL_DIFFERENTIATION) | 15 | 0.035 | 0.069 | 1550 | tags=87%, list=50%, signal=172% |
| 79 | [POSITIVE_REGULATION_OF_I_KAPPAB_KINASE_NF_KAPPAB_CASCADE](http://www.broadinstitute.org/gsea/msigdb/cards/POSITIVE_REGULATION_OF_I_KAPPAB_KINASE_NF_KAPPAB_CASCADE) | 21 | 0.037 | 0.089 | 1581 | tags=81%, list=51%, signal=164% |
| 83 | [NEGATIVE_REGULATION_OF_TRANSCRIPTION_DNA_DEPENDENT](http://www.broadinstitute.org/gsea/msigdb/cards/NEGATIVE_REGULATION_OF_TRANSCRIPTION_DNA_DEPENDENT) | 33 | 0.040 | 0.089 | 1629 | tags=76%, list=52%, signal=158% |
| 85 | [CELLULAR_MACROMOLECULE_METABOLIC_PROCESS](http://www.broadinstitute.org/gsea/msigdb/cards/CELLULAR_MACROMOLECULE_METABOLIC_PROCESS) | 216 | 0.040 | 0.096 | 2102 | tags=77%, list=68%, signal=222% |
| 81 | [TRANSLATION](http://www.broadinstitute.org/gsea/msigdb/cards/TRANSLATION) | 18 | 0.041 | 0.088 | 2076 | tags=100%, list=67%, signal=300% |
| 82 | [POST_TRANSLATIONAL_PROTEIN_MODIFICATION](http://www.broadinstitute.org/gsea/msigdb/cards/POST_TRANSLATIONAL_PROTEIN_MODIFICATION) | 108 | 0.043 | 0.089 | 2092 | tags=81%, list=67%, signal=239% |
| 87 | [DNA_BINDING](http://www.broadinstitute.org/gsea/msigdb/cards/DNA_BINDING) | 138 | 0.043 | 0.097 | 1872 | tags=72%, list=60%, signal=173% |
| 89 | [CELL_PROLIFERATION_GO_0008283](http://www.broadinstitute.org/gsea/msigdb/cards/CELL_PROLIFERATION_GO_0008283) | 117 | 0.045 | 0.103 | 2059 | tags=79%, list=66%, signal=225% |
| 92 | [ANTI_APOPTOSIS](http://www.broadinstitute.org/gsea/msigdb/cards/ANTI_APOPTOSIS) | 22 | 0.046 | 0.120 | 1845 | tags=86%, list=59%, signal=212% |
| 90 | [INFLAMMATORY_RESPONSE](http://www.broadinstitute.org/gsea/msigdb/cards/INFLAMMATORY_RESPONSE) | 23 | 0.047 | 0.116 | 624 | tags=48%, list=20%, signal=59% |
| 94 | [PROTEIN_COMPLEX_ASSEMBLY](http://www.broadinstitute.org/gsea/msigdb/cards/PROTEIN_COMPLEX_ASSEMBLY) | 47 | 0.049 | 0.121 | 2135 | tags=87%, list=69%, signal=275% |
| 95 | [CHROMOSOME_ORGANIZATION_AND_BIOGENESIS](http://www.broadinstitute.org/gsea/msigdb/cards/CHROMOSOME_ORGANIZATION_AND_BIOGENESIS) | 29 | 0.050 | 0.121 | 1845 | tags=83%, list=59%, signal=202% |
| 78 | [NEGATIVE_REGULATION_OF_NUCLEOBASENUCLEOSIDENUCLEOTIDE_AND_NUCLEIC_ACID_METABOLIC_PROCESS](http://www.broadinstitute.org/gsea/msigdb/cards/NEGATIVE_REGULATION_OF_NUCLEOBASENUCLEOSIDENUCLEOTIDE_AND_NUCLEIC_ACID_METABOLIC_PROCESS) | 51 | 0.051 | 0.087 | 2131 | tags=88%, list=69%, signal=277% |
| 91 | [ACTIN_CYTOSKELETON_ORGANIZATION_AND_BIOGENESIS](http://www.broadinstitute.org/gsea/msigdb/cards/ACTIN_CYTOSKELETON_ORGANIZATION_AND_BIOGENESIS) | 30 | 0.052 | 0.117 | 2047 | tags=90%, list=66%, signal=262% |
| 86 | [RAS_PROTEIN_SIGNAL_TRANSDUCTION](http://www.broadinstitute.org/gsea/msigdb/cards/RAS_PROTEIN_SIGNAL_TRANSDUCTION) | 18 | 0.053 | 0.098 | 1792 | tags=89%, list=58%, signal=209% |
| 88 | [NEGATIVE_REGULATION_OF_RNA_METABOLIC_PROCESS](http://www.broadinstitute.org/gsea/msigdb/cards/NEGATIVE_REGULATION_OF_RNA_METABOLIC_PROCESS) | 33 | 0.054 | 0.099 | 1629 | tags=76%, list=52%, signal=158% |
| 97 | [TRANSCRIPTION_ACTIVATOR_ACTIVITY](http://www.broadinstitute.org/gsea/msigdb/cards/TRANSCRIPTION_ACTIVATOR_ACTIVITY) | 35 | 0.056 | 0.124 | 1629 | tags=74%, list=52%, signal=155% |
| 96 | [MICROTUBULE_BASED_PROCESS](http://www.broadinstitute.org/gsea/msigdb/cards/MICROTUBULE_BASED_PROCESS) | 25 | 0.058 | 0.124 | 2053 | tags=92%, list=66%, signal=270% |
| 101 | NUCLEAR_LUMEN | 58 | 0.059 | 0.126 | 2156 | tags=86%, list=69%, signal=277% |
| 100 | [MULTICELLULAR_ORGANISMAL_DEVELOPMENT](http://www.broadinstitute.org/gsea/msigdb/cards/MULTICELLULAR_ORGANISMAL_DEVELOPMENT) | 202 | 0.063 | 0.126 | 630 | tags=29%, list=20%, signal=34% |
| 98 | [POSITIVE_REGULATION_OF_RNA_METABOLIC_PROCESS](http://www.broadinstitute.org/gsea/msigdb/cards/POSITIVE_REGULATION_OF_RNA_METABOLIC_PROCESS) | 31 | 0.064 | 0.125 | 1699 | tags=77%, list=55%, signal=169% |
| 93 | [CELLULAR_PROTEIN_METABOLIC_PROCESS](http://www.broadinstitute.org/gsea/msigdb/cards/CELLULAR_PROTEIN_METABOLIC_PROCESS) | 213 | 0.065 | 0.122 | 2102 | tags=77%, list=68%, signal=221% |
| 106 | ENZYME_LINKED_RECEPTOR_PROTEIN_SIGNALING_PATHWAY | 32 | 0.066 | 0.138 | 1080 | tags=56%, list=35%, signal=85% |
| 99 | [PROTEIN_TYROSINE_PHOSPHATASE_ACTIVITY](http://www.broadinstitute.org/gsea/msigdb/cards/PROTEIN_TYROSINE_PHOSPHATASE_ACTIVITY) | 17 | 0.068 | 0.127 | 709 | tags=53%, list=23%, signal=68% |
| 103 | ACTIN_FILAMENT_BASED_PROCESS | 33 | 0.078 | 0.131 | 1754 | tags=79%, list=57%, signal=179% |
| 105 | PHOSPHOPROTEIN_PHOSPHATASE_ACTIVITY | 22 | 0.078 | 0.137 | 1441 | tags=73%, list=46%, signal=135% |
| 107 | G_PROTEIN_COUPLED_RECEPTOR_ACTIVITY | 18 | 0.085 | 0.138 | 645 | tags=50%, list=21%, signal=63% |
| 104 | INTRACELLULAR_TRANSPORT | 52 | 0.086 | 0.130 | 2021 | tags=83%, list=65%, signal=233% |
| 102 | TRANSMEMBRANE_RECEPTOR_ACTIVITY | 54 | 0.089 | 0.128 | 493 | tags=33%, list=16%, signal=39% |
| 108 | POSITIVE_REGULATION_OF_TRANSCRIPTIONDNA_DEPENDENT | 30 | 0.089 | 0.154 | 2002 | tags=87%, list=65%, signal=242% |
| 110 | CALCIUM_ION_BINDING | 26 | 0.095 | 0.169 | 355 | tags=35%, list=11%, signal=39% |
| 111 | SMALL_GTPASE_REGULATOR_ACTIVITY | 17 | 0.097 | 0.175 | 1852 | tags=88%, list=60%, signal=218% |
| 109 | REGULATION_OF_CELL_PROLIFERATION | 59 | 0.098 | 0.153 | 1765 | tags=73%, list=57%, signal=166% |
| 115 | REGULATION_OF_TRANSCRIPTION_FROM_RNA_POLYMERASE_II_PROMOTER | 68 | 0.110 | 0.200 | 1758 | tags=71%, list=57%, signal=159% |
| 114 | INTRACELLULAR_PROTEIN_TRANSPORT | 28 | 0.116 | 0.187 | 1963 | tags=86%, list=63%, signal=231% |
| 112 | NEGATIVE_REGULATION_OF_TRANSCRIPTION_FROM_RNA_POLYMERASE_II_PROMOTER | 19 | 0.119 | 0.182 | 1628 | tags=79%, list=52%, signal=165% |
| 113 | PROTEIN_TRANSPORT | 28 | 0.122 | 0.187 | 1963 | tags=86%, list=63%, signal=231% |
| 116 | RECEPTOR_SIGNALING_PROTEIN_ACTIVITY | 21 | 0.128 | 0.207 | 2023 | tags=90%, list=65%, signal=258% |
| 117 | ENZYME_ACTIVATOR_ACTIVITY | 29 | 0.129 | 0.217 | 1917 | tags=83%, list=62%, signal=215% |
| 121 | MUSCLE_DEVELOPMENT | 21 | 0.138 | 0.238 | 1165 | tags=62%, list=38%, signal=98% |
| 118 | BIOPOLYMER_CATABOLIC_PROCESS | 24 | 0.139 | 0.230 | 1874 | tags=83%, list=60%, signal=209% |
| 119 | TRANSMEMBRANE_RECEPTOR_PROTEIN_TYROSINE_KINASE_SIGNALING_PATHWAY | 19 | 0.140 | 0.230 | 1649 | tags=79%, list=53%, signal=167% |
| 120 | ENZYME_REGULATOR_ACTIVITY | 77 | 0.150 | 0.234 | 2138 | tags=82%, list=69%, signal=257% |
| 124 | REGULATION_OF_MOLECULAR_FUNCTION | 69 | 0.168 | 0.257 | 2065 | tags=80%, list=67%, signal=233% |
| 122 | NEGATIVE_REGULATION_OF_CELL_PROLIFERATION | 33 | 0.178 | 0.259 | 1765 | tags=76%, list=57%, signal=174% |
| 125 | PROTEIN_TARGETING | 21 | 0.179 | 0.279 | 1963 | tags=86%, list=63%, signal=232% |
| 123 | RNA_BINDING | 25 | 0.185 | 0.258 | 2442 | tags=100%, list=79%, signal=466% |
| 133 | PHOSPHORIC_MONOESTER_HYDROLASE_ACTIVITY | 31 | 0.201 | 0.331 | 1441 | tags=65%, list=46%, signal=119% |
| 126 | PROTEIN_AMINO_ACID_DEPHOSPHORYLATION | 17 | 0.206 | 0.296 | 1959 | tags=88%, list=63%, signal=238% |
| 127 | NEGATIVE_REGULATION_OF_CELL_CYCLE | 26 | 0.207 | 0.297 | 1746 | tags=77%, list=56%, signal=174% |
| 130 | ACTIN_BINDING | 19 | 0.207 | 0.330 | 1574 | tags=74%, list=51%, signal=149% |
| 128 | PROTEIN_AMINO_ACID_PHOSPHORYLATION | 69 | 0.208 | 0.311 | 1502 | tags=61%, list=48%, signal=115% |
| 129 | CELL_JUNCTION | 17 | 0.220 | 0.311 | 1969 | tags=88%, list=63%, signal=240% |
| 131 | RECEPTOR_ACTIVITY | 86 | 0.234 | 0.335 | 493 | tags=27%, list=16%, signal=31% |
| 132 | GTPASE_REGULATOR_ACTIVITY | 36 | 0.239 | 0.333 | 1889 | tags=78%, list=61%, signal=196% |
| 139 | PROTEIN_MODIFICATION_PROCESS | 134 | 0.243 | 0.363 | 2380 | tags=85%, list=77%, signal=349% |
| 134 | POSITIVE_REGULATION_OF_TRANSFERASE_ACTIVITY | 18 | 0.248 | 0.330 | 1520 | tags=72%, list=49%, signal=141% |
| 137 | CELL_SURFACE_RECEPTOR_LINKED_SIGNAL_TRANSDUCTION_GO_0007166 | 91 | 0.249 | 0.357 | 1523 | tags=59%, list=49%, signal=113% |
| 135 | SMALL_GTPASE_MEDIATED_SIGNAL_TRANSDUCTION | 23 | 0.251 | 0.341 | 2047 | tags=87%, list=66%, signal=254% |
| 140 | REGULATION_OF_CATALYTIC_ACTIVITY | 59 | 0.254 | 0.363 | 1765 | tags=69%, list=57%, signal=158% |
| 136 | PROTEIN_DIMERIZATION_ACTIVITY | 49 | 0.262 | 0.359 | 1970 | tags=78%, list=63%, signal=209% |
| 141 | PROTEIN_C_TERMINUS_BINDING | 20 | 0.264 | 0.366 | 1953 | tags=85%, list=63%, signal=228% |
| 146 | ESTABLISHMENT_OF_PROTEIN_LOCALIZATION | 35 | 0.293 | 0.419 | 2447 | tags=94%, list=79%, signal=441% |
| 138 | POSITIVE_REGULATION_OF_CATALYTIC_ACTIVITY | 31 | 0.296 | 0.361 | 1742 | tags=74%, list=56%, signal=167% |
| 143 | RESPONSE_TO_CHEMICAL_STIMULUS | 66 | 0.306 | 0.399 | 953 | tags=42%, list=31%, signal=60% |
| 142 | ESTABLISHMENT_OF_CELLULAR_LOCALIZATION | 71 | 0.315 | 0.400 | 2045 | tags=77%, list=66%, signal=222% |
| 144 | PHOSPHORYLATION | 78 | 0.315 | 0.403 | 1502 | tags=59%, list=48%, signal=111% |
| 145 | GUANYL_NUCLEOTIDE_EXCHANGE_FACTOR_ACTIVITY | 17 | 0.316 | 0.418 | 1852 | tags=82%, list=60%, signal=203% |
| 147 | NUCLEAR_TRANSPORT | 18 | 0.333 | 0.417 | 2447 | tags=100%, list=79%, signal=470% |
| 148 | DEPHOSPHORYLATION | 19 | 0.336 | 0.427 | 1959 | tags=84%, list=63%, signal=227% |
| 150 | NUCLEOCYTOPLASMIC_TRANSPORT | 18 | 0.341 | 0.438 | 2447 | tags=100%, list=79%, signal=470% |
| 152 | REGULATION_OF_MAP_KINASE_ACTIVITY | 19 | 0.342 | 0.442 | 681 | tags=42%, list=22%, signal=54% |
| 151 | REGULATION_OF_BIOLOGICAL_QUALITY | 77 | 0.347 | 0.436 | 2004 | tags=75%, list=65%, signal=207% |
| 153 | SECOND_MESSENGER_MEDIATED_SIGNALING | 15 | 0.348 | 0.449 | 739 | tags=47%, list=24%, signal=61% |
| 149 | REGULATION_OF_HYDROLASE_ACTIVITY | 15 | 0.357 | 0.439 | 1754 | tags=80%, list=57%, signal=183% |
| 157 | TRANSCRIPTION_COFACTOR_ACTIVITY | 56 | 0.364 | 0.462 | 2128 | tags=80%, list=69%, signal=251% |
| 154 | SECRETION | 32 | 0.368 | 0.452 | 1742 | tags=72%, list=56%, signal=162% |
| 156 | GROWTH | 16 | 0.390 | 0.459 | 1444 | tags=69%, list=47%, signal=128% |
| 159 | SOLUBLE_FRACTION | 24 | 0.392 | 0.493 | 1666 | tags=71%, list=54%, signal=152% |
| 158 | PROTEIN_KINASE_BINDING | 18 | 0.393 | 0.484 | 2135 | tags=89%, list=69%, signal=283% |
| 155 | BIOPOLYMER_MODIFICATION | 140 | 0.399 | 0.462 | 2380 | tags=84%, list=77%, signal=345% |
| 161 | STRUCTURAL_MOLECULE_ACTIVITY | 35 | 0.414 | 0.497 | 2122 | tags=83%, list=68%, signal=259% |
| 160 | KINASE_BINDING | 18 | 0.420 | 0.493 | 2135 | tags=89%, list=69%, signal=283% |
| 162 | REGULATION_OF_CELLULAR_COMPONENT_ORGANIZATION_AND_BIOGENESIS | 27 | 0.431 | 0.500 | 2144 | tags=85%, list=69%, signal=273% |
| 166 | PROTEIN_BINDING_BRIDGING | 17 | 0.455 | 0.550 | 1947 | tags=82%, list=63%, signal=220% |
| 163 | REGULATION_OF_MULTICELLULAR_ORGANISMAL_PROCESS | 27 | 0.457 | 0.522 | 875 | tags=44%, list=28%, signal=61% |
| 164 | CELL_PROJECTION | 24 | 0.460 | 0.535 | 502 | tags=33%, list=16%, signal=39% |
| 171 | REGULATION_OF_PROTEIN_KINASE_ACTIVITY | 39 | 0.461 | 0.579 | 1193 | tags=51%, list=38%, signal=82% |
| 167 | LEADING_EDGE | 15 | 0.474 | 0.549 | 2047 | tags=87%, list=66%, signal=253% |
| 165 | MACROMOLECULE_LOCALIZATION | 45 | 0.488 | 0.552 | 2447 | tags=91%, list=79%, signal=425% |
| 168 | ENZYME_BINDING | 50 | 0.500 | 0.566 | 2249 | tags=84%, list=72%, signal=300% |
| 169 | REGULATION_OF_TRANSFERASE_ACTIVITY | 39 | 0.504 | 0.572 | 1193 | tags=51%, list=38%, signal=82% |
| 170 | TRANSCRIPTION_COREPRESSOR_ACTIVITY | 24 | 0.517 | 0.578 | 1181 | tags=54%, list=38%, signal=87% |
| 173 | RESPONSE_TO_STRESS | 108 | 0.532 | 0.591 | 168 | tags=13%, list=5%, signal=13% |
| 172 | GTPASE_ACTIVATOR_ACTIVITY | 16 | 0.536 | 0.592 | 1722 | tags=75%, list=55%, signal=168% |
| 175 | REGULATION_OF_KINASE_ACTIVITY | 39 | 0.540 | 0.604 | 1193 | tags=51%, list=38%, signal=82% |
| 174 | TRANSCRIPTION_COACTIVATOR_ACTIVITY | 25 | 0.545 | 0.599 | 2242 | tags=88%, list=72%, signal=315% |
| 178 | VASCULATURE_DEVELOPMENT | 16 | 0.554 | 0.619 | 1747 | tags=75%, list=56%, signal=171% |
| 176 | IDENTICAL_PROTEIN_BINDING | 76 | 0.567 | 0.615 | 2296 | tags=83%, list=74%, signal=311% |
| 177 | HEMOPOIETIC_OR_LYMPHOID_ORGAN_DEVELOPMENT | 15 | 0.585 | 0.617 | 649 | tags=40%, list=21%, signal=50% |
| 179 | CELL_CYCLE_ARREST_GO_0007050 | 18 | 0.596 | 0.641 | 2037 | tags=83%, list=66%, signal=241% |
| 180 | PROTEIN_SERINE_THREONINE_KINASE_ACTIVITY | 51 | 0.598 | 0.639 | 1502 | tags=59%, list=48%, signal=112% |
| 181 | MACROMOLECULE_BIOSYNTHETIC_PROCESS | 40 | 0.614 | 0.641 | 2120 | tags=80%, list=68%, signal=249% |
| 182 | HYDROLASE_ACTIVITY_ACTING_ON_ESTER_BONDS | 52 | 0.669 | 0.707 | 709 | tags=33%, list=23%, signal=42% |
| 184 | PROTEIN_LOCALIZATION | 40 | 0.673 | 0.701 | 2447 | tags=90%, list=79%, signal=420% |
| 183 | PROTEIN_HOMODIMERIZATION_ACTIVITY | 33 | 0.706 | 0.704 | 1970 | tags=76%, list=63%, signal=205% |
| 185 | IMMUNE_SYSTEM_DEVELOPMENT | 16 | 0.707 | 0.727 | 649 | tags=38%, list=21%, signal=47% |
| 186 | CELL_CELL_ADHESION | 15 | 0.747 | 0.787 | 1970 | tags=80%, list=63%, signal=218% |
| 187 | RNA_POLYMERASE_II_TRANSCRIPTION_FACTOR_ACTIVITY | 32 | 0.774 | 0.784 | 1686 | tags=66%, list=54%, signal=142% |
| 188 | ENDOPEPTIDASE_ACTIVITY | 19 | 0.792 | 0.823 | 864 | tags=42%, list=28%, signal=58% |
| 189 | CELL_SURFACE | 18 | 0.824 | 0.835 | 77 | tags=17%, list=2%, signal=17% |
| 190 | ACTIN_CYTOSKELETON | 37 | 0.846 | 0.851 | 1879 | tags=70%, list=61%, signal=176% |
| 191 | PHOSPHORIC_ESTER_HYDROLASE_ACTIVITY | 36 | 0.877 | 0.903 | 1441 | tags=56%, list=46%, signal=103% |
| 192 | PEPTIDASE_ACTIVITY | 31 | 0.893 | 0.925 | 2713 | tags=97%, list=87%, signal=762% |
| 193 | PURINE_RIBONUCLEOTIDE_BINDING | 53 | 0.930 | 0.938 | 1539 | tags=57%, list=50%, signal=110% |
