# Supplementary material for: Host cell transcriptome modification upon exogenous HPV16 L2 protein expression
Source: Oncotarget. 2017 Oct 12;8(53):90730–47. doi: 10.18632/oncotarget.21817 (PMC5710881; doi:10.18632/oncotarget.21817)
Supplement: Supplementary file 6 [file oncotarget-08-90730-s006.docx]

**Table S5. Gene Set Enrichment Analysis for negatively regulated gene sets in L2h_8fwb comparison.**

|  | **Gene Sets follow link to MSigDB** | **SIZE** | **NOM**  **p-val** | **FDR**  **q-val** | **RANK AT MAX** | **LEADING EDGE** |
| --- | --- | --- | --- | --- | --- | --- |
| 1 | [CYTOPLASMIC_PART](http://www.broadinstitute.org/gsea/msigdb/cards/CYTOPLASMIC_PART) | 275 | 0.000 | 0.000 | 1740 | tags=79%, list=56%, signal=163% |
| 2 | [CYTOPLASM](http://www.broadinstitute.org/gsea/msigdb/cards/CYTOPLASM) | 426 | 0.000 | 0.000 | 1758 | tags=72%, list=57%, signal=143% |
| 3 | [MITOCHONDRION](http://www.broadinstitute.org/gsea/msigdb/cards/MITOCHONDRION) | 65 | 0.000 | 0.000 | 1628 | tags=92%, list=52%, signal=190% |
| 4 | [ORGANELLE_PART](http://www.broadinstitute.org/gsea/msigdb/cards/ORGANELLE_PART) | 233 | 0.000 | 0.000 | 1820 | tags=78%, list=59%, signal=175% |
| 5 | [OXIDOREDUCTASE_ACTIVITY](http://www.broadinstitute.org/gsea/msigdb/cards/OXIDOREDUCTASE_ACTIVITY) | 73 | 0.000 | 0.000 | 1387 | tags=79%, list=45%, signal=140% |
| 6 | [INTRACELLULAR_ORGANELLE_PART](http://www.broadinstitute.org/gsea/msigdb/cards/INTRACELLULAR_ORGANELLE_PART) | 232 | 0.000 | 0.000 | 1820 | tags=78%, list=59%, signal=175% |
| 7 | [MACROMOLECULAR_COMPLEX](http://www.broadinstitute.org/gsea/msigdb/cards/MACROMOLECULAR_COMPLEX) | 178 | 0.000 | 0.000 | 1772 | tags=76%, list=57%, signal=168% |
| 8 | [ORGANELLE_MEMBRANE](http://www.broadinstitute.org/gsea/msigdb/cards/ORGANELLE_MEMBRANE) | 62 | 0.000 | 0.000 | 1820 | tags=90%, list=59%, signal=214% |
| 9 | [PROTEIN_COMPLEX](http://www.broadinstitute.org/gsea/msigdb/cards/PROTEIN_COMPLEX) | 166 | 0.000 | 0.000 | 1772 | tags=77%, list=57%, signal=169% |
| 10 | [OXIDOREDUCTASE_ACTIVITY_GO_0016616](http://www.broadinstitute.org/gsea/msigdb/cards/OXIDOREDUCTASE_ACTIVITY_GO_0016616) | 21 | 0.000 | 0.000 | 1296 | tags=95%, list=42%, signal=162% |
| 11 | [OXIDOREDUCTASE_ACTIVITY_ACTING_ON_CH_OH_GROUP_OF_DONORS](http://www.broadinstitute.org/gsea/msigdb/cards/OXIDOREDUCTASE_ACTIVITY_ACTING_ON_CH_OH_GROUP_OF_DONORS) | 21 | 0.000 | 0.000 | 1296 | tags=95%, list=42%, signal=162% |
| 12 | [CARBOXYLIC_ACID_METABOLIC_PROCESS](http://www.broadinstitute.org/gsea/msigdb/cards/CARBOXYLIC_ACID_METABOLIC_PROCESS) | 43 | 0.000 | 0.000 | 1553 | tags=86%, list=50%, signal=170% |
| 13 | [MITOCHONDRIAL_PART](http://www.broadinstitute.org/gsea/msigdb/cards/MITOCHONDRIAL_PART) | 23 | 0.000 | 0.000 | 1588 | tags=100%, list=51%,signal=203% |
| 14 | [ENVELOPE](http://www.broadinstitute.org/gsea/msigdb/cards/ENVELOPE) | 37 | 0.000 | 0.000 | 1588 | tags=89%, list=51%, signal=180% |
| 15 | [ORGANIC_ACID_METABOLIC_PROCESS](http://www.broadinstitute.org/gsea/msigdb/cards/ORGANIC_ACID_METABOLIC_PROCESS) | 43 | 0.000 | 0.000 | 1553 | tags=86%, list=50%, signal=170% |
| 16 | [ORGANELLE_ENVELOPE](http://www.broadinstitute.org/gsea/msigdb/cards/ORGANELLE_ENVELOPE) | 37 | 0.000 | 0.000 | 1588 | tags=89%, list=51%, signal=180% |
| 17 | [CELL_CYCLE_PROCESS](http://www.broadinstitute.org/gsea/msigdb/cards/CELL_CYCLE_PROCESS) | 59 | 0.000 | 0.000 | 1653 | tags=83%, list=53%, signal=174% |
| 18 | [MONOCARBOXYLIC_ACID_METABOLIC_PROCESS](http://www.broadinstitute.org/gsea/msigdb/cards/MONOCARBOXYLIC_ACID_METABOLIC_PROCESS) | 19 | 0.000 | 0.000 | 1385 | tags=95%, list=45%, signal=170% |
| 19 | [COFACTOR_METABOLIC_PROCESS](http://www.broadinstitute.org/gsea/msigdb/cards/COFACTOR_METABOLIC_PROCESS) | 17 | 0.000 | 0.000 | 1456 | tags=100%, list=47%,signal=187% |
| 20 | [M_PHASE](http://www.broadinstitute.org/gsea/msigdb/cards/M_PHASE) | 35 | 0.000 | 0.000 | 1611 | tags=89%, list=52%, signal=182% |
| 21 | [MITOCHONDRIAL_ENVELOPE](http://www.broadinstitute.org/gsea/msigdb/cards/MITOCHONDRIAL_ENVELOPE) | 20 | 0.000 | 0.001 | 1588 | tags=100%, list=51%,signal=203% |
| 22 | [CELL_CYCLE_PHASE](http://www.broadinstitute.org/gsea/msigdb/cards/CELL_CYCLE_PHASE) | 53 | 0.000 | 0.001 | 1653 | tags=83%, list=53%, signal=175% |
| 23 | [ENDOMEMBRANE_SYSTEM](http://www.broadinstitute.org/gsea/msigdb/cards/ENDOMEMBRANE_SYSTEM) | 44 | 0.000 | 0.001 | 1820 | tags=91%, list=59%, signal=217% |
| 24 | [MITOCHONDRIAL_MEMBRANE](http://www.broadinstitute.org/gsea/msigdb/cards/MITOCHONDRIAL_MEMBRANE) | 17 | 0.000 | 0.001 | 1588 | tags=100%, list=51%,signal=204% |
| 25 | [ELECTRON_CARRIER_ACTIVITY](http://www.broadinstitute.org/gsea/msigdb/cards/ELECTRON_CARRIER_ACTIVITY) | 24 | 0.000 | 0.003 | 1347 | tags=83%, list=43%, signal=146% |
| 26 | [M_PHASE_OF_MITOTIC_CELL_CYCLE](http://www.broadinstitute.org/gsea/msigdb/cards/M_PHASE_OF_MITOTIC_CELL_CYCLE) | 31 | 0.000 | 0.003 | 1611 | tags=87%, list=52%, signal=179% |
| 28 | [GENERATION_OF_PRECURSOR_METABOLITES_AND_ENERGY](http://www.broadinstitute.org/gsea/msigdb/cards/GENERATION_OF_PRECURSOR_METABOLITES_AND_ENERGY) | 34 | 0.000 | 0.006 | 1637 | tags=85%, list=53%, signal=179% |
| 29 | [MITOSIS](http://www.broadinstitute.org/gsea/msigdb/cards/MITOSIS) | 29 | 0.000 | 0.006 | 1611 | tags=86%, list=52%, signal=178% |
| 30 | [LIPID_METABOLIC_PROCESS](http://www.broadinstitute.org/gsea/msigdb/cards/LIPID_METABOLIC_PROCESS) | 60 | 0.000 | 0.007 | 1047 | tags=58%, list=34%, signal=86% |
| 32 | [CELLULAR_LIPID_METABOLIC_PROCESS](http://www.broadinstitute.org/gsea/msigdb/cards/CELLULAR_LIPID_METABOLIC_PROCESS) | 46 | 0.000 | 0.008 | 1508 | tags=76%, list=49%, signal=146% |
| 34 | [NERVOUS_SYSTEM_DEVELOPMENT](http://www.broadinstitute.org/gsea/msigdb/cards/NERVOUS_SYSTEM_DEVELOPMENT) | 59 | 0.000 | 0.015 | 439 | tags=37%, list=14%, signal=43% |
| 31 | [ORGANELLE_INNER_MEMBRANE](http://www.broadinstitute.org/gsea/msigdb/cards/ORGANELLE_INNER_MEMBRANE) | 17 | 0.002 | 0.007 | 1524 | tags=94%, list=49%, signal=184% |
| 33 | [CELL_CYCLE_GO_0007049](http://www.broadinstitute.org/gsea/msigdb/cards/CELL_CYCLE_GO_0007049) | 95 | 0.002 | 0.011 | 2236 | tags=91%, list=72%, signal=314% |
| 41 | [MICROTUBULE_ORGANIZING_CENTER](http://www.broadinstitute.org/gsea/msigdb/cards/MICROTUBULE_ORGANIZING_CENTER) | 18 | 0.002 | 0.034 | 1600 | tags=89%, list=52%, signal=182% |
| 27 | [ENDOPLASMIC_RETICULUM](http://www.broadinstitute.org/gsea/msigdb/cards/ENDOPLASMIC_RETICULUM) | 56 | 0.004 | 0.005 | 1758 | tags=82%, list=57%, signal=186% |
| 35 | [DNA_METABOLIC_PROCESS](http://www.broadinstitute.org/gsea/msigdb/cards/DNA_METABOLIC_PROCESS) | 55 | 0.004 | 0.016 | 1599 | tags=75%, list=52%, signal=151% |
| 36 | [INTRINSIC_TO_MEMBRANE](http://www.broadinstitute.org/gsea/msigdb/cards/INTRINSIC_TO_MEMBRANE) | 214 | 0.004 | 0.018 | 531 | tags=29%, list=17%, signal=32% |
| 37 | [MICROTUBULE_CYTOSKELETON](http://www.broadinstitute.org/gsea/msigdb/cards/MICROTUBULE_CYTOSKELETON) | 46 | 0.004 | 0.019 | 1600 | tags=76%, list=52%, signal=155% |
| 40 | [INTEGRAL_TO_MEMBRANE](http://www.broadinstitute.org/gsea/msigdb/cards/INTEGRAL_TO_MEMBRANE) | 210 | 0.004 | 0.034 | 526 | tags=28%, list=17%, signal=32% |
| 38 | [INTRINSIC_TO_PLASMA_MEMBRANE](http://www.broadinstitute.org/gsea/msigdb/cards/INTRINSIC_TO_PLASMA_MEMBRANE) | 153 | 0.008 | 0.031 | 546 | tags=31%, list=18%, signal=35% |
| 42 | [PYROPHOSPHATASE_ACTIVITY](http://www.broadinstitute.org/gsea/msigdb/cards/PYROPHOSPHATASE_ACTIVITY) | 53 | 0.008 | 0.036 | 1705 | tags=77%, list=55%, signal=169% |
| 43 | [ESTABLISHMENT_OF_LOCALIZATION](http://www.broadinstitute.org/gsea/msigdb/cards/ESTABLISHMENT_OF_LOCALIZATION) | 167 | 0.008 | 0.036 | 1821 | tags=71%, list=59%, signal=162% |
| 45 | [BIOSYNTHETIC_PROCESS](http://www.broadinstitute.org/gsea/msigdb/cards/BIOSYNTHETIC_PROCESS) | 83 | 0.008 | 0.039 | 1711 | tags=72%, list=55%, signal=157% |
| 52 | [DNA_REPAIR](http://www.broadinstitute.org/gsea/msigdb/cards/DNA_REPAIR) | 30 | 0.010 | 0.052 | 1727 | tags=83%, list=56%, signal=186% |
| 39 | [HYDROLASE_ACTIVITY_ACTING_ON_ACID_ANHYDRIDES](http://www.broadinstitute.org/gsea/msigdb/cards/HYDROLASE_ACTIVITY_ACTING_ON_ACID_ANHYDRIDES) | 53 | 0.011 | 0.032 | 1705 | tags=77%, list=55%, signal=169% |
| 53 | [NUCLEOSIDE_TRIPHOSPHATASE_ACTIVITY](http://www.broadinstitute.org/gsea/msigdb/cards/NUCLEOSIDE_TRIPHOSPHATASE_ACTIVITY) | 51 | 0.012 | 0.053 | 1705 | tags=76%, list=55%, signal=167% |
| 44 | [INTEGRAL_TO_PLASMA_MEMBRANE](http://www.broadinstitute.org/gsea/msigdb/cards/INTEGRAL_TO_PLASMA_MEMBRANE) | 152 | 0.013 | 0.037 | 546 | tags=30%, list=18%, signal=35% |
| 48 | [CATABOLIC_PROCESS](http://www.broadinstitute.org/gsea/msigdb/cards/CATABOLIC_PROCESS) | 46 | 0.013 | 0.043 | 1662 | tags=76%, list=54%, signal=161% |
| 46 | [MITOTIC_CELL_CYCLE](http://www.broadinstitute.org/gsea/msigdb/cards/MITOTIC_CELL_CYCLE) | 54 | 0.014 | 0.040 | 1970 | tags=85%, list=63%, signal=229% |
| 49 | [CELLULAR_CATABOLIC_PROCESS](http://www.broadinstitute.org/gsea/msigdb/cards/CELLULAR_CATABOLIC_PROCESS) | 44 | 0.014 | 0.045 | 1662 | tags=77%, list=54%, signal=164% |
| 50 | [RESPONSE_TO_DNA_DAMAGE_STIMULUS](http://www.broadinstitute.org/gsea/msigdb/cards/RESPONSE_TO_DNA_DAMAGE_STIMULUS) | 38 | 0.014 | 0.047 | 1750 | tags=82%, list=56%, signal=185% |
| 58 | [ELECTRON_TRANSPORT_GO_0006118](http://www.broadinstitute.org/gsea/msigdb/cards/ELECTRON_TRANSPORT_GO_0006118) | 22 | 0.014 | 0.072 | 1454 | tags=77%, list=47%, signal=144% |
| 62 | [TRANSFERASE_ACTIVITY_TRANSFERRING_ACYL_GROUPS](http://www.broadinstitute.org/gsea/msigdb/cards/TRANSFERASE_ACTIVITY_TRANSFERRING_ACYL_GROUPS) | 16 | 0.014 | 0.078 | 1650 | tags=88%, list=53%, signal=186% |
| 55 | [SUBSTRATE_SPECIFIC_TRANSMEMBRANE_TRANSPORTER_ACTIVITY](http://www.broadinstitute.org/gsea/msigdb/cards/SUBSTRATE_SPECIFIC_TRANSMEMBRANE_TRANSPORTER_ACTIVITY) | 53 | 0.018 | 0.057 | 1002 | tags=53%, list=32%, signal=77% |
| 51 | [SUBSTRATE_SPECIFIC_TRANSPORTER_ACTIVITY](http://www.broadinstitute.org/gsea/msigdb/cards/SUBSTRATE_SPECIFIC_TRANSPORTER_ACTIVITY) | 64 | 0.020 | 0.049 | 1002 | tags=52%, list=32%, signal=75% |
| 57 | [CYTOSOL](http://www.broadinstitute.org/gsea/msigdb/cards/CYTOSOL) | 43 | 0.020 | 0.068 | 1704 | tags=77%, list=55%, signal=168% |
| 47 | [CHROMOSOMAL_PART](http://www.broadinstitute.org/gsea/msigdb/cards/CHROMOSOMAL_PART) | 31 | 0.021 | 0.042 | 1951 | tags=90%, list=63%, signal=241% |
| 65 | [CARBOHYDRATE_METABOLIC_PROCESS](http://www.broadinstitute.org/gsea/msigdb/cards/CARBOHYDRATE_METABOLIC_PROCESS) | 37 | 0.023 | 0.081 | 1711 | tags=78%, list=55%, signal=173% |
| 59 | [ANATOMICAL_STRUCTURE_DEVELOPMENT](http://www.broadinstitute.org/gsea/msigdb/cards/ANATOMICAL_STRUCTURE_DEVELOPMENT) | 197 | 0.025 | 0.071 | 511 | tags=26%, list=16%, signal=30% |
| 60 | [CELLULAR_CARBOHYDRATE_METABOLIC_PROCESS](http://www.broadinstitute.org/gsea/msigdb/cards/CELLULAR_CARBOHYDRATE_METABOLIC_PROCESS) | 31 | 0.026 | 0.079 | 1711 | tags=81%, list=55%, signal=178% |
| 66 | [CATION_TRANSPORT](http://www.broadinstitute.org/gsea/msigdb/cards/CATION_TRANSPORT) | 19 | 0.026 | 0.080 | 965 | tags=63%, list=31%, signal=91% |
| 54 | [CHROMOSOME](http://www.broadinstitute.org/gsea/msigdb/cards/CHROMOSOME) | 37 | 0.027 | 0.057 | 1758 | tags=81%, list=57%, signal=185% |
| 64 | [TRANSMEMBRANE_TRANSPORTER_ACTIVITY](http://www.broadinstitute.org/gsea/msigdb/cards/TRANSMEMBRANE_TRANSPORTER_ACTIVITY) | 59 | 0.027 | 0.080 | 1002 | tags=51%, list=32%, signal=74% |
| 56 | [LIPID_BIOSYNTHETIC_PROCESS](http://www.broadinstitute.org/gsea/msigdb/cards/LIPID_BIOSYNTHETIC_PROCESS) | 25 | 0.030 | 0.068 | 1183 | tags=68%, list=38%, signal=109% |
| 61 | [TRANSPORT](http://www.broadinstitute.org/gsea/msigdb/cards/TRANSPORT) | 145 | 0.030 | 0.078 | 1821 | tags=70%, list=59%, signal=162% |
| 67 | [NON_MEMBRANE_BOUND_ORGANELLE](http://www.broadinstitute.org/gsea/msigdb/cards/NON_MEMBRANE_BOUND_ORGANELLE) | 144 | 0.030 | 0.084 | 2206 | tags=83%, list=71%, signal=273% |
| 63 | [CYTOSKELETAL_PART](http://www.broadinstitute.org/gsea/msigdb/cards/CYTOSKELETAL_PART) | 65 | 0.031 | 0.081 | 2229 | tags=89%, list=72%, signal=310% |
| 69 | [RESPONSE_TO_ENDOGENOUS_STIMULUS](http://www.broadinstitute.org/gsea/msigdb/cards/RESPONSE_TO_ENDOGENOUS_STIMULUS) | 48 | 0.034 | 0.086 | 1750 | tags=77%, list=56%, signal=174% |
| 71 | [NEUROLOGICAL_SYSTEM_PROCESS](http://www.broadinstitute.org/gsea/msigdb/cards/NEUROLOGICAL_SYSTEM_PROCESS) | 40 | 0.034 | 0.091 | 1197 | tags=60%, list=39%, signal=96% |
| 68 | [INTRACELLULAR_NON_MEMBRANE_BOUND_ORGANELLE](http://www.broadinstitute.org/gsea/msigdb/cards/INTRACELLULAR_NON_MEMBRANE_BOUND_ORGANELLE) | 144 | 0.036 | 0.084 | 2206 | tags=83%, list=71%, signal=273% |
| 77 | [MEMBRANE_BOUND_VESICLE](http://www.broadinstitute.org/gsea/msigdb/cards/MEMBRANE_BOUND_VESICLE) | 26 | 0.036 | 0.109 | 1704 | tags=81%, list=55%, signal=178% |
| 70 | [NUCLEAR_ENVELOPE_ENDOPLASMIC_RETICULUM_NETWORK](http://www.broadinstitute.org/gsea/msigdb/cards/NUCLEAR_ENVELOPE_ENDOPLASMIC_RETICULUM_NETWORK) | 18 | 0.037 | 0.092 | 1758 | tags=89%, list=57%, signal=204% |
| 75 | [PLASMA_MEMBRANE_PART](http://www.broadinstitute.org/gsea/msigdb/cards/PLASMA_MEMBRANE_PART) | 184 | 0.039 | 0.109 | 526 | tags=27%, list=17%, signal=30% |
| 72 | [GOLGI_APPARATUS](http://www.broadinstitute.org/gsea/msigdb/cards/GOLGI_APPARATUS) | 53 | 0.040 | 0.095 | 1821 | tags=77%, list=59%, signal=184% |
| 74 | [REGULATION_OF_MITOSIS](http://www.broadinstitute.org/gsea/msigdb/cards/REGULATION_OF_MITOSIS) | 17 | 0.041 | 0.104 | 1750 | tags=88%, list=56%, signal=201% |
| 76 | [CELL_CYCLE_CHECKPOINT_GO_0000075](http://www.broadinstitute.org/gsea/msigdb/cards/CELL_CYCLE_CHECKPOINT_GO_0000075) | 17 | 0.041 | 0.109 | 1750 | tags=88%, list=56%, signal=201% |
| 73 | [CENTROSOME](http://www.broadinstitute.org/gsea/msigdb/cards/CENTROSOME) | 15 | 0.044 | 0.094 | 1600 | tags=87%, list=52%, signal=178% |
| 83 | [SUBSTRATE_SPECIFIC_CHANNEL_ACTIVITY](http://www.broadinstitute.org/gsea/msigdb/cards/SUBSTRATE_SPECIFIC_CHANNEL_ACTIVITY) | 18 | 0.045 | 0.130 | 965 | tags=61%, list=31%, signal=88% |
| 81 | [ATPASE_ACTIVITY](http://www.broadinstitute.org/gsea/msigdb/cards/ATPASE_ACTIVITY) | 30 | 0.048 | 0.128 | 1950 | tags=87%, list=63%, signal=231% |
| 80 | [SYSTEM_PROCESS](http://www.broadinstitute.org/gsea/msigdb/cards/SYSTEM_PROCESS) | 72 | 0.049 | 0.115 | 1197 | tags=54%, list=39%, signal=86% |
| 78 | [MEMBRANE](http://www.broadinstitute.org/gsea/msigdb/cards/MEMBRANE) | 354 | 0.050 | 0.108 | 1800 | tags=65%, list=58%, signal=137% |
| 87 | [TRANSFERASE_ACTIVITY_TRANSFERRING_PHOSPHORUS_CONTAINING_GROUPS](http://www.broadinstitute.org/gsea/msigdb/cards/TRANSFERASE_ACTIVITY_TRANSFERRING_PHOSPHORUS_CONTAINING_GROUPS) | 107 | 0.058 | 0.143 | 2092 | tags=79%, list=67%, signal=235% |
| 82 | [SYSTEM_DEVELOPMENT](http://www.broadinstitute.org/gsea/msigdb/cards/SYSTEM_DEVELOPMENT) | 159 | 0.061 | 0.128 | 511 | tags=26%, list=16%, signal=30% |
| 79 | [DNA_DEPENDENT_DNA_REPLICATION](http://www.broadinstitute.org/gsea/msigdb/cards/DNA_DEPENDENT_DNA_REPLICATION) | 16 | 0.068 | 0.116 | 1705 | tags=88%, list=55%, signal=193% |
| 85 | [CELLULAR_BIOSYNTHETIC_PROCESS](http://www.broadinstitute.org/gsea/msigdb/cards/CELLULAR_BIOSYNTHETIC_PROCESS) | 50 | 0.069 | 0.145 | 1553 | tags=68%, list=50%, signal=134% |
| 88 | [VESICLE](http://www.broadinstitute.org/gsea/msigdb/cards/VESICLE) | 27 | 0.072 | 0.157 | 1704 | tags=78%, list=55%, signal=171% |
| 92 | [REGULATION_OF_CELL_CYCLE](http://www.broadinstitute.org/gsea/msigdb/cards/REGULATION_OF_CELL_CYCLE) | 58 | 0.076 | 0.167 | 2236 | tags=88%, list=72%, signal=309% |
| 84 | [CYTOPLASMIC_MEMBRANE_BOUND_VESICLE](http://www.broadinstitute.org/gsea/msigdb/cards/CYTOPLASMIC_MEMBRANE_BOUND_VESICLE) | 25 | 0.078 | 0.147 | 1945 | tags=88%, list=63%, signal=234% |
| 96 | [ION_TRANSPORT](http://www.broadinstitute.org/gsea/msigdb/cards/ION_TRANSPORT) | 23 | 0.084 | 0.179 | 965 | tags=57%, list=31%, signal=81% |
| 86 | [NUCLEAR_ENVELOPE](http://www.broadinstitute.org/gsea/msigdb/cards/NUCLEAR_ENVELOPE) | 17 | 0.086 | 0.145 | 1792 | tags=88%, list=58%, signal=208% |
| 91 | [ENDOPLASMIC_RETICULUM_PART](http://www.broadinstitute.org/gsea/msigdb/cards/ENDOPLASMIC_RETICULUM_PART) | 19 | 0.088 | 0.167 | 1740 | tags=84%, list=56%, signal=191% |
| 90 | [DNA_REPLICATION](http://www.broadinstitute.org/gsea/msigdb/cards/DNA_REPLICATION) | 25 | 0.090 | 0.164 | 1574 | tags=76%, list=51%, signal=153% |
| 94 | [AMINO_ACID_AND_DERIVATIVE_METABOLIC_PROCESS](http://www.broadinstitute.org/gsea/msigdb/cards/AMINO_ACID_AND_DERIVATIVE_METABOLIC_PROCESS) | 21 | 0.090 | 0.181 | 1553 | tags=76%, list=50%, signal=151% |
| 95 | [MEMBRANE_PART](http://www.broadinstitute.org/gsea/msigdb/cards/MEMBRANE_PART) | 279 | 0.090 | 0.180 | 1686 | tags=61%, list=54%, signal=122% |
| 100 | [PLASMA_MEMBRANE](http://www.broadinstitute.org/gsea/msigdb/cards/PLASMA_MEMBRANE) | 234 | 0.092 | 0.200 | 526 | tags=24%, list=17%, signal=27% |
| 89 | [ENDOPLASMIC_RETICULUM_MEMBRANE](http://www.broadinstitute.org/gsea/msigdb/cards/ENDOPLASMIC_RETICULUM_MEMBRANE) | 15 | 0.097 | 0.165 | 1713 | tags=87%, list=55%, signal=193% |
| 93 | [METAL_ION_TRANSPORT](http://www.broadinstitute.org/gsea/msigdb/cards/METAL_ION_TRANSPORT) | 17 | 0.107 | 0.180 | 577 | tags=47%, list=19%, signal=57% |
| 102 | GATED_CHANNEL_ACTIVITY | 15 | 0.108 | 0.204 | 965 | tags=60%, list=31%, signal=87% |
| 98 | [MACROMOLECULE_CATABOLIC_PROCESS](http://www.broadinstitute.org/gsea/msigdb/cards/MACROMOLECULE_CATABOLIC_PROCESS) | 28 | 0.111 | 0.198 | 1662 | tags=75%, list=54%, signal=160% |
| 99 | [ION_CHANNEL_ACTIVITY](http://www.broadinstitute.org/gsea/msigdb/cards/ION_CHANNEL_ACTIVITY) | 17 | 0.116 | 0.198 | 965 | tags=59%, list=31%, signal=85% |
| 97 | [AMINO_ACID_METABOLIC_PROCESS](http://www.broadinstitute.org/gsea/msigdb/cards/AMINO_ACID_METABOLIC_PROCESS) | 18 | 0.121 | 0.196 | 1553 | tags=78%, list=50%, signal=155% |
| 101 | MEMBRANE_FRACTION | 77 | 0.123 | 0.200 | 437 | tags=27%, list=14%, signal=31% |
| 105 | NITROGEN_COMPOUND_METABOLIC_PROCESS | 31 | 0.124 | 0.207 | 1553 | tags=71%, list=50%, signal=141% |
| 104 | ACTIVE_TRANSMEMBRANE_TRANSPORTER_ACTIVITY | 32 | 0.125 | 0.202 | 1692 | tags=75%, list=55%, signal=163% |
| 108 | KINASE_ACTIVITY | 96 | 0.128 | 0.217 | 2483 | tags=92%, list=80%, signal=445% |
| 107 | GTPASE_ACTIVITY | 21 | 0.133 | 0.218 | 1608 | tags=76%, list=52%, signal=157% |
| 109 | TRANSMISSION_OF_NERVE_IMPULSE | 17 | 0.134 | 0.242 | 1186 | tags=65%, list=38%, signal=104% |
| 103 | CELLULAR_MACROMOLECULE_CATABOLIC_PROCESS | 25 | 0.136 | 0.203 | 1662 | tags=76%, list=54%, signal=162% |
| 106 | NUCLEOPLASM | 48 | 0.136 | 0.207 | 2272 | tags=90%, list=73%, signal=329% |
| 110 | SYNAPTIC_TRANSMISSION | 17 | 0.138 | 0.242 | 1186 | tags=65%, list=38%, signal=104% |
| 112 | PROTEIN_CATABOLIC_PROCESS | 17 | 0.140 | 0.242 | 2303 | tags=100%, list=74%,signal=386% |
| 113 | CYTOPLASMIC_VESICLE | 26 | 0.145 | 0.247 | 1704 | tags=77%, list=55%, signal=169% |
| 114 | ATPASE_ACTIVITY_COUPLED | 21 | 0.154 | 0.275 | 1950 | tags=86%, list=63%, signal=229% |
| 118 | ANATOMICAL_STRUCTURE_MORPHOGENESIS | 80 | 0.163 | 0.277 | 488 | tags=28%, list=16%, signal=32% |
| 111 | RESPONSE_TO_ABIOTIC_STIMULUS | 27 | 0.168 | 0.244 | 1750 | tags=78%, list=56%, signal=177% |
| 116 | ESTABLISHMENT_AND_OR_MAINTENANCE_OF_CHROMATIN_ARCHITECTURE | 16 | 0.176 | 0.277 | 2096 | tags=94%, list=68%, signal=287% |
| 117 | CELLULAR_PROTEIN_CATABOLIC_PROCESS | 15 | 0.182 | 0.279 | 1634 | tags=80%, list=53%, signal=168% |
| 119 | NUCLEOPLASM_PART | 38 | 0.186 | 0.302 | 2596 | tags=100%, list=84%,signal=605% |
| 115 | INTERPHASE | 24 | 0.187 | 0.277 | 1653 | tags=75%, list=53%, signal=159% |
| 120 | CELLULAR_COMPONENT_ASSEMBLY | 60 | 0.215 | 0.328 | 2078 | tags=80%, list=67%, signal=238% |
| 122 | PROTEINACEOUS_EXTRACELLULAR_MATRIX | 18 | 0.222 | 0.343 | 491 | tags=39%, list=16%, signal=46% |
| 123 | EXTRACELLULAR_MATRIX | 18 | 0.234 | 0.341 | 491 | tags=39%, list=16%, signal=46% |
| 121 | INTERPHASE_OF_MITOTIC_CELL_CYCLE | 23 | 0.235 | 0.337 | 1653 | tags=74%, list=53%, signal=157% |
| 124 | PHOSPHOTRANSFERASE_ACTIVITY_ALCOHOL_GROUP_AS_ACCEPTOR | 86 | 0.240 | 0.364 | 2483 | tags=91%, list=80%, signal=441% |
| 127 | MAPKKK_CASCADE_GO_0000165 | 28 | 0.248 | 0.372 | 539 | tags=36%, list=17%, signal=43% |
| 125 | CYTOSKELETON | 102 | 0.263 | 0.368 | 2229 | tags=81%, list=72%, signal=279% |
| 126 | SENSORY_PERCEPTION | 24 | 0.265 | 0.374 | 1197 | tags=58%, list=39%, signal=94% |
| 132 | LIGASE_ACTIVITY | 19 | 0.276 | 0.396 | 1634 | tags=74%, list=53%, signal=155% |
| 129 | CELLULAR_LOCALIZATION | 73 | 0.282 | 0.397 | 2071 | tags=78%, list=67%, signal=229% |
| 130 | ALCOHOL_METABOLIC_PROCESS | 20 | 0.299 | 0.394 | 1656 | tags=75%, list=53%, signal=160% |
| 133 | ION_TRANSMEMBRANE_TRANSPORTER_ACTIVITY | 38 | 0.299 | 0.408 | 1002 | tags=47%, list=32%, signal=69% |
| 128 | VESICLE_MEDIATED_TRANSPORT | 41 | 0.305 | 0.393 | 2345 | tags=90%, list=76%, signal=365% |
| 134 | ION_BINDING | 72 | 0.310 | 0.406 | 1202 | tags=50%, list=39%, signal=80% |
| 131 | CATION_CHANNEL_ACTIVITY | 15 | 0.313 | 0.399 | 305 | tags=33%, list=10%, signal=37% |
| 135 | GOLGI_APPARATUS_PART | 16 | 0.313 | 0.428 | 1820 | tags=81%, list=59%, signal=195% |
| 137 | MACROMOLECULAR_COMPLEX_ASSEMBLY | 57 | 0.324 | 0.443 | 2078 | tags=79%, list=67%, signal=235% |
| 136 | CELLULAR_HOMEOSTASIS | 20 | 0.326 | 0.443 | 618 | tags=40%, list=20%, signal=50% |
| 138 | CATION_BINDING | 55 | 0.329 | 0.443 | 1202 | tags=51%, list=39%, signal=82% |
| 139 | PROTEIN_HETERODIMERIZATION_ACTIVITY | 20 | 0.342 | 0.451 | 2024 | tags=85%, list=65%, signal=243% |
| 143 | AMINE_METABOLIC_PROCESS | 27 | 0.383 | 0.500 | 1553 | tags=67%, list=50%, signal=132% |
| 140 | RESPONSE_TO_RADIATION | 20 | 0.392 | 0.478 | 1880 | tags=80%, list=61%, signal=202% |
| 145 | METAL_ION_TRANSMEMBRANE_TRANSPORTER_ACTIVITY | 22 | 0.408 | 0.529 | 305 | tags=27%, list=10%, signal=30% |
| 142 | MOLECULAR_ADAPTOR_ACTIVITY | 15 | 0.412 | 0.502 | 2427 | tags=100%, list=78%,signal=457% |
| 144 | LIPID_BINDING | 16 | 0.412 | 0.511 | 2049 | tags=88%, list=66%, signal=256% |
| 141 | CATION_HOMEOSTASIS | 15 | 0.419 | 0.486 | 546 | tags=40%, list=18%, signal=48% |
| 148 | TRANSITION_METAL_ION_BINDING | 29 | 0.440 | 0.562 | 1139 | tags=52%, list=37%, signal=81% |
| 147 | G_PROTEIN_COUPLED_RECEPTOR_PROTEIN_SIGNALING_PATHWAY | 34 | 0.448 | 0.560 | 656 | tags=35%, list=21%, signal=44% |
| 146 | CHEMICAL_HOMEOSTASIS | 23 | 0.461 | 0.554 | 546 | tags=35%, list=18%, signal=42% |
| 150 | GLYCOPROTEIN_BIOSYNTHETIC_PROCESS | 17 | 0.461 | 0.560 | 1942 | tags=82%, list=63%, signal=219% |
| 149 | MEMBRANE_LIPID_METABOLIC_PROCESS | 19 | 0.488 | 0.563 | 1047 | tags=53%, list=34%, signal=79% |
| 151 | MEMBRANE_ORGANIZATION_AND_BIOGENESIS | 28 | 0.496 | 0.576 | 2202 | tags=86%, list=71%, signal=293% |
| 152 | GLYCOPROTEIN_METABOLIC_PROCESS | 20 | 0.502 | 0.584 | 1942 | tags=80%, list=63%, signal=212% |
| 154 | CELL_FRACTION | 102 | 0.505 | 0.627 | 437 | tags=22%, list=14%, signal=24% |
| 153 | PROTEIN_KINASE_ACTIVITY | 68 | 0.516 | 0.584 | 2483 | tags=90%, list=80%, signal=439% |
| 155 | REGULATION_OF_TRANSPORT | 15 | 0.560 | 0.628 | 2285 | tags=93%, list=74%, signal=352% |
| 156 | NEGATIVE_REGULATION_OF_CATALYTIC_ACTIVITY | 15 | 0.565 | 0.663 | 2504 | tags=100%, list=81%,signal=516% |
| 157 | MAGNESIUM_ION_BINDING | 19 | 0.566 | 0.662 | 2092 | tags=84%, list=67%, signal=257% |
| 158 | HOMEOSTATIC_PROCESS | 34 | 0.605 | 0.699 | 618 | tags=32%, list=20%, signal=40% |
| 159 | ADENYL_NUCLEOTIDE_BINDING | 47 | 0.666 | 0.763 | 2198 | tags=81%, list=71%, signal=273% |
| 160 | PROTEOLYSIS | 41 | 0.695 | 0.776 | 1634 | tags=63%, list=53%, signal=132% |
| 161 | CYTOSKELETAL_PROTEIN_BINDING | 38 | 0.710 | 0.781 | 2028 | tags=76%, list=65%, signal=218% |
| 162 | CATION_TRANSMEMBRANE_TRANSPORTER_ACTIVITY | 33 | 0.714 | 0.776 | 305 | tags=21%, list=10%, signal=23% |
| 163 | ZINC_ION_BINDING | 22 | 0.715 | 0.786 | 838 | tags=41%, list=27%, signal=56% |
| 164 | KINASE_REGULATOR_ACTIVITY | 16 | 0.739 | 0.810 | 2215 | tags=88%, list=71%, signal=304% |
| 167 | ORGAN_MORPHOGENESIS | 33 | 0.749 | 0.834 | 1535 | tags=61%, list=49%, signal=119% |
| 166 | SECRETION_BY_CELL | 20 | 0.754 | 0.814 | 2038 | tags=80%, list=66%, signal=232% |
| 169 | PROTEIN_KINASE_REGULATOR_ACTIVITY | 15 | 0.788 | 0.831 | 134 | tags=20%, list=4%, signal=21% |
| 165 | SKELETAL_DEVELOPMENT | 20 | 0.790 | 0.815 | 339 | tags=25%, list=11%, signal=28% |
| 168 | ENZYME_INHIBITOR_ACTIVITY | 23 | 0.790 | 0.832 | 278 | tags=22%, list=9%, signal=24% |
| 170 | ADENYL_RIBONUCLEOTIDE_BINDING | 45 | 0.801 | 0.851 | 2198 | tags=80%, list=71%, signal=270% |
| 172 | RESPONSE_TO_LIGHT_STIMULUS | 16 | 0.813 | 0.854 | 1489 | tags=63%, list=48%, signal=120% |
| 171 | ION_HOMEOSTASIS | 19 | 0.823 | 0.846 | 546 | tags=32%, list=18%, signal=38% |
| 174 | PEPTIDYL_AMINO_ACID_MODIFICATION | 15 | 0.880 | 0.927 | 1850 | tags=73%, list=60%, signal=181% |
| 173 | TRANSFERASE_ACTIVITY_TRANSFERRING_GLYCOSYL_GROUPS | 26 | 0.903 | 0.924 | 1942 | tags=73%, list=63%, signal=194% |
| 175 | TRANSFERASE_ACTIVITY_TRANSFERRING_HEXOSYL_GROUPS | 21 | 0.908 | 0.932 | 1711 | tags=67%, list=55%, signal=148% |
| 177 | PURINE_NUCLEOTIDE_BINDING | 55 | 0.924 | 0.942 | 2890 | tags=100%, list=93%, signal=1431% |
| 176 | NUCLEOTIDE_BINDING | 57 | 0.925 | 0.942 | 2890 | tags=100%, list=93%, signal=1430% |
| 178 | ATP_BINDING | 41 | 0.929 | 0.940 | 2703 | tags=95%, list=87%, signal=728% |
