# Supplementary material for: Host cell transcriptome modification upon exogenous HPV16 L2 protein expression
Source: Oncotarget. 2017 Oct 12;8(53):90730–47. doi: 10.18632/oncotarget.21817 (PMC5710881; doi:10.18632/oncotarget.21817)
Supplement: Supplementary file 7 [file oncotarget-08-90730-s007.docx]

**Table S6.** Top 50 genes in combined ranking list of SVM and RF.

| **SVM Rank** | **RF Rank** | **Combination of SVM and RF** | **Gene Symbol** |
| --- | --- | --- | --- |
| 5 | 17 | 5.5 | PMEPA1 |
| 41 | 7 | 12 | ZNF608 |
| 2 | 70 | 18 | SNX10 |
| 4 | 70 | 18.5 | HSPB3 |
| 8 | 70 | 19.5 | OLR1 |
| 75 | 7 | 20.5 | FA2H |
| 24 | 70 | 23.5 | PHGR1 |
| 27 | 70 | 24.25 | SPTA1 |
| 37 | 70 | 26.75 | PTPRG |
| 94 | 13 | 26.75 | KLHDC7B |
| 40 | 70 | 27.5 | MYEOV |
| 42 | 70 | 28 | APCDD1 |
| 111 | 1 | 28 | S100A7 |
| 48 | 70 | 29.5 | ARL14 |
| 53 | 70 | 30.75 | C3 |
| 57 | 70 | 31.75 | SDPR |
| 58 | 70 | 32 | ABCA5 |
| 60 | 70 | 32.5 | LCN2 |
| 64 | 70 | 33.5 | RAB26 |
| 71 | 70 | 35.25 | LCE5A |
| 72 | 70 | 35.5 | MUC4 |
| 91 | 70 | 40.25 | DHRS9 |
| 92 | 70 | 40.5 | ZFP57 |
| 93 | 70 | 40.75 | SLC7A11 |
| 95 | 70 | 41.25 | SERPINB3 |
| 98 | 70 | 42 | FLG |
| 105 | 70 | 43.75 | TMEM56 |
| 112 | 70 | 45.5 | SIRPB2 |
| 175 | 7 | 45.5 | NEURL3 |
| 116 | 70 | 46.5 | IFI44L |
| 117 | 70 | 46.75 | PSG5 |
| 122 | 70 | 48 | SEMA4A |
| 186 | 7 | 48.25 | AKR1C3 |
| 124 | 70 | 48.5 | BATF2 |
| 125 | 70 | 48.75 | LY6G6C |
| 129 | 70 | 49.75 | FGF13 |
| 133 | 70 | 50.75 | SRCRB4D |
| 142 | 70 | 53 | LGALSL |
| 202 | 17 | 54.75 | TCN1 |
| 218 | 7 | 56.25 | CAMK4 |
| 155 | 70 | 56.25 | PTPLAD2 |
| 161 | 70 | 57.75 | TTC9 |
| 163 | 70 | 58.25 | APOLD1 |
| 174 | 70 | 61 | ACTBL2 |
| 180 | 70 | 62.5 | CD274 |
| 183 | 70 | 63.25 | PARD6B |
| 234 | 21 | 63.75 | ABCA4 |
| 191 | 70 | 65.25 | AGPAT4 |
| 196 | 70 | 66.5 | FAM171A1 |
| 203 | 70 | 68.25 | NGFR |
